# MreB filaments in the elongasome modulate *E. coli* membrane curvature

**DOI:** 10.1016/j.bpj.2025.08.036

**Published:** 2025-09-02

**Authors:** Becca W.A. Baileeves, Anthony D.Q. Hoang, Timothy D.H. Bugg, Phillip J. Stansfeld

**Affiliations:** 1 School of Life Sciences, University of Warwick, Coventry, United Kingdom; 2 MRC DTP, Warwick Medical School, University of Warwick, Coventry, United Kingdom; 3 Department of Chemistry, University of Warwick, Coventry, United Kingdom

## Abstract

MreB, a bacterial actin homolog, plays a pivotal role in defining the shape of rod-shaped bacteria by coordinating peptidoglycan synthesis during cell elongation. It forms filaments that interact with the cytosolic leaflet of the cell membrane, as well as with membrane proteins and other cytosolic proteins. In this study, molecular dynamics simulations were used to investigate the interactions between *Escherichia coli* (*E. coli*) MreB and model cytoplasmic membranes. The simulations reveal that MreB filaments recruit cardiolipin and induce membrane bending toward the periplasmic space. Cardiolipin exhibits a concentration-dependent effect on bending, highlighting its critical role in this mechanism. Simulations with MreB mutants identify residues R105 and R136 as key contributors to both cardiolipin recruitment and membrane bending. Additionally, removal of the N-terminal helix in *E. coli* MreB was shown to reduce membrane bending. This study concludes that MreB induces membrane bending through two distinct mechanisms: 1) recruitment of the cone-shaped lipid cardiolipin and 2) physical distortion by the N-terminal helix. These findings uncover a novel mechanism by which MreB alters membrane architecture, offering insights into how other proteins are recruited to the filament in the *E. coli* elongasome.

## Significance

Antimicrobial resistance is a consistently growing global health burden. An improved understanding of essential processes in bacteria will aid in future antibiotic development. Peptidoglycan is an essential component of bacterial cell walls. Most rod-shaped bacteria use MreB as part of the coordination of peptidoglycan synthesis during cell elongation. This study uses molecular dynamics simulations to uncover interactions between MreB and the lipid membrane. Specifically, the lipid cardiolipin is recruited to MreB binding sites and results in a localized bending of the membrane. In silico mutagenesis and modifications to the membrane composition aim to elucidate the mechanism of membrane bending. The observed modification of the membrane by MreB may play a role in MreB dynamics and/or recruitment of peptidoglycan synthesis enzymes.

## Introduction

The peptidoglycan is an essential component of bacterial cell walls that protects cells from osmotic pressure inside the cells, preventing cells from bursting. This essentiality and its specificity to bacteria make the peptidoglycan and its synthesis a popular target for antibiotics. Many enzymes work together to build the peptidoglycan in a stepwise process.

MreB is a bacterial actin homolog found in all rod-shaped bacteria with a key role in determining cell shape (1,2). MreB forms antiparallel double filaments that cycle around the periphery of the cell, powered by peptidoglycan synthesis (3,4,5). This cell-shape protein coordinates peptidoglycan synthesis during cell elongation through interactions with enzymes RodA and PBP2, which attach new peptidoglycan units from substrate lipid II to the growing peptidoglycan strands. These enzymes are part of a complex named the elongasome; it grows peptidoglycan at the appropriate site and orientation to maintain the rod shape of cells, oriented by MreB (3,4,5,6,7). Additional cell-shape proteins MreC and MreD are also part of the elongasome, and disrupting MreB, C, or D disrupts cell shape and decreases cell viability (8,9). MreB plays this coordinating role in most rod-shaped bacteria, including four of the six ESKAPE pathogens, which have been deemed high-risk pathogens for their rapid acquiring of resistance to antibiotics (10,11). There are exceptions of specific rod-shaped bacteria that grow at the poles of the cell such as *Mycobacterium tuberculosis*, which do not contain genes for MreB (12,13).

Despite evidence for interactions between various proteins in the elongasome, the overall structure of this protein complex is unknown, as well as its interactions with the membrane. It is difficult to determine the structure of such a large complex experimentally, although components have been solved including the interactions between RodA and PBP2 (14,15). In this paper, computational methods are used to begin to elucidate interactions at the elongasome. Here, we focus primarily on interactions of MreB with the membrane, using molecular dynamics (MD) simulations to study these interactions. We observe preferential binding of MreB to cardiolipin (CL) over other lipids in the membrane and simultaneous bending of the membrane perpendicular to the length of MreB. Peptidoglycan precursor lipid II is also recruited to MreB filaments when included in the membrane. These previously unobserved interactions may play a role in the recruitment of membrane proteins involved in peptidoglycan synthesis to MreB filaments, or the dynamics of this complex.

Experimental evidence has previously shown the importance of the N-terminal helix and a spatially close hydrophobic loop in binding of MreB to membranes (16,17). In this study, we predict these regions of the protein as having a role in CL recruitment and membrane bending. Further residues, particularly anionic ones, were identified as important in MreB-membrane interactions through computational mutagenesis studies. Finally in this study, elongasome simulations are set up with a continuous MreB filament, to confirm that the observed results occur in a more “native” state of MreB and the membrane.

## Materials and methods

### Protein structural models

Protein structures from *E. coli* were predicted using AlphaFold 2 (18). These are expected to be accurate as they align well with experimental structures of different homologs of MreB (backbone RMSD compared with PDB: 4CZI = 1.514 Å, 7ZPT = 2.994 Å, and IJCF = 1.796 Å). MreB dimer structures from AlphaFold 2 were aligned in PyMOL (19) to a dimer within the MreB hexamer so that they are in a conformation that can be repeated to form filaments.

MreB mutants were produced using the PyMOL mutagenesis tool. The secondary structure of the mutants was compared with WT by running 3 × 250 ns atomistic simulations of each mutant and running DSSP analysis (Fig. S1). Overall, the secondary structure propensity remains similar in the mutants. The N-terminal helix does not remain as a helix for the entire simulation in the WT, either in solution or membrane bound, making it difficult to compare to the mutants, which also adopt either an alpha-helix or loop at the N-terminal helix. However, we assumed a helical structure for the N-terminal helix in the coarse-grained simulations.

An AlphaFold 3 model with CL bound to MreB is also included. Here, a local version of AlphaFold 3 was used (20).

### Molecular dynamics simulations

The protein models were coarse-grained to the Martini3 (21) description using “martinize2.” Where present, the orientation of proteins in the membrane was predicted using MemEmbed (22). Simplified *E. coli* membranes (23) were generated using Insane (24) and contained 70% phosphatidylethanolamine (PE), 20% phosphatidylglycerol (PG), and 10% CL unless stated otherwise. In simulations of MreB monomers or dimers associating to the membrane, a box size of 14 × 14 × 20 nm was used, and the protein translated 10 nm away from the center of the membrane. A box size of y axis 30 nm and z axis 21 nm was used across all systems with filaments. Box x axis size was determined by the length of the MreB filaments, around 20 nm for filaments containing four dimer units. MreB filaments were translated by 6 nm in the z axis to place them out of but close to the membrane. To make the filaments “continuous,” an elastic network was formed across the periodic boundary between one end of the filament and the other end.

Energy minimization, equilibration, and finally the MD simulations were run in GROMACS (25). Energy minimization was performed on the protein alone and then (for each of five repeats) the whole system, each time for 5000 steps. Equilibration was 10,000 steps of 20 fs each. During the equilibration, Berendsen semiisotropic pressure coupling was used with time constant 12 ps, compressibility 3e−4 bar^−1^, and reference pressure of 1 bar. V-rescale temperature coupling was applied separately to the protein, lipid, and solvent (including ions), with time constant 1 ps and reference temperature of 310 K for each. To ensure that an equilibrated state was reached, bending analysis of the subsequent MD run was performed at each 1-μs interval in the WT system with 70% PE, 20% PG, and 10% CL. An ANOVA did not reveal any significant difference between time points (*p* = 0.513), so we consider the equilibration time to be sufficient.

The MD simulations were all 5 μs with 20-fs steps, for each of the five repeats. Here, Berendsen anisotropic pressure coupling was used for all systems containing continuous MreB filaments so that the box can expand in the y axis while the x axis stays the same size due to the filament. Systems without continuous MreB filaments use the more accurate C-rescale barostat with semiisotropic pressure coupling; this barostat can be used with semiisotropic coupling but not anisotropic. All systems had a pressure coupling time constant of 12 ps. Anisotropic systems had compressibility values of 3e−4 bar^−1^ and reference pressure 1 bar for the xx, yy, and zz components, and 0 bar^−1^ or 0 bar respectively for all other components. Semiisotropic systems (Berendsen or C-rescale) had compressibility of 3e−4 bar^−1^ and reference pressure of 1 bar. Temperature coupling was the same as in the equilibration for all simulations.

Alternative parameters based on a recent paper were also tested, due to a concern about artificial membrane distortions (26). Here, in the parameters for the simulation run, nstlist was changed to 20 (previously 10) and verlet-buffer-tolerance to 0.0002 (previously 0.005), and rlist = 1.35 was added (previously undefined, so a default of 1 was used). The membrane bending was similar with the new parameters, so the previously defined parameters remain used in all other simulations (Fig. S2).

### Lipid concentrations

The lipid concentrations used were defined when running the Insane script to set up the membrane (24). Symmetrical membranes were used so the lipid ratios are equal in each leaflet of the membrane. CL, containing four lipid tails, is larger than PE and PG, which contain two lipid tails. The area per lipid of PG, PE, and CL was measured by setting up membranes with 100% PE, PG, or CL, simulating them for 10 ns in semiisotropic conditions, calculating the area of the resulting membrane, and dividing this by the number of lipids in each leaflet. The resulting area per lipid values were 0.63 nm^2^ for PE, 0.67 nm^2^ for PG, and 1.23 nm^2^ for CL. To avoid instability in membranes with higher CL concentrations, the ratio of lipids was used to calculate the appropriate area per lipid for each system. For example, in a 70% PE, 20% PG, and 10% CL membrane, average area per lipid is (0.7 × 0.63) + (0.2 × 0.67) + (0.1 × 1.23) = 0.698 nm^2^ (rounded to 0.70 nm^2^). Following this experiment, future 10% CL membranes were set up with an area per lipid of 0.7 nm^2^ as opposed to the default 0.6 nm^2^. Only semiisotropic systems such as the nonfilament MreB-membrane association simulations were set up with an area per lipid of 0.6 nm^2^, and here the box expanded during equilibration to allow for a thermodynamically stable membrane.

In one set of simulations, a neutral CL is used as a control (not biologically relevant) to investigate the role of the shape of CL. Here, the Q5 beads with −1 charge in the phosphate headgroup of CL were changed to P5 beads with 0 charge.

### Elongasome MD simulations

AlphaFold 2 multimer was used to produce a model of the elongasome containing six copies of the MreB monomer and one copy each of MreC, MreD, RodA, PBP2, and RodZ, all from *E. coli*. This model was then aligned to the continuous MreB filament used in the previous simulations. The protein models were coarse-grained to the Martini3 (21) description using “martinize2,” MemEmbed (22) was used to predict the position of the membrane and orientation of the elongasome, and Insane (24) was used to build the membrane. A box size of y axis 30 nm and z axis 27 nm was used, with the size of the x axis determined by the length of the filament, around 20 nm. Energy minimization and equilibration were performed as described in molecular dynamics simulations methods above.

AlphaFold2-multimer was used to produce a model of the elongasome containing six copies of the MreB monomer and one copy each of MreC, MreD, RodA, PBP2, and RodZ, all from *E. coli*. This model was then aligned to the continuous MreB filament used in the previous simulations. MemEmbed was used to predict the position of the membrane and orientation of the elongasome, the proteins were converted to a Martini3 coarse-grained representation, and Insane was used to build the membrane. A box size of y axis 30 nm and z axis 27 nm was used, with the size of the x axis determined by the length of the filament, approximately 20 nm. Energy minimization and equilibration were performed as described above.

### Analysis

Interactions between lipids and protein were measured using PyLipID (27), using a dual cutoff scheme of 0.475 nm and 0.8 nm. This means that an interaction is considered to begin when the distance between coarse-grained beads is less than 0.475 nm and ends when the distance is greater than 0.8 nm.

For lipid density and membrane bending plots, PLUMED (28) was used to measure the positions of the specified lipid or protein beads. For lipid positions, the phosphate bead from the lipid headgroup (or one of the phosphates in the case of CL) was used to represent the position of that lipid. In 2D lipid density plots, normalized density is between 0 and 1 from zero density to maximum density, plotted as a 2D histogram with 30 bins per axis. In 1D lipid density plots, normalized density is calculated as lipids per MreB dimer per frame per bin. The bin refers to the histogram bins, and because in all cases the size of the box is the same on the y axis and the same number of bins are used, all bins are size 1.3 Å on the y axis. For example, if there are four dimers in an MreB filament, 5000 frames in the simulation data, and over the course of the simulation the lipid CL was found in the 1.3 Å bin on the y axis 2000 times, the normalized density would be 2000/(4 × 5000) = 0.1.

Lipid enrichment measurements were calculated by defining the y position of the different peaks and troughs of the lipid density and getting a count for the total number of the specified lipid in that y position. Y positions were defined as shown in Figs. S8–S10. A lipid enrichment measure was calculated for each of five repeats, for each of the mutants or different conditions. Statistical tests were carried out using the scipy.stats module in Python as follows: a Shapiro-Wilk test for normality, one-way ANOVA, and then Tukey-HSD.

Membrane bending plots were generated by calculating the mean z-position of the lipid phosphate group from the inner leaflet of the membrane, using a bin size of 1 Å. Z-position was normalized by subtracting the average z-position at the periphery of the plotted y values. Standard error of the mean was also calculated and plotted. Membrane bending was quantified as the maximum normalized z-position. A bending value was calculated per repeat, and statistical tests were carried out as in the lipid enrichment measurements.

All plots were produced in Python, and molecular visualization was performed in PyMOL or VMD (29).

## Results

### MreB preferentially binds cardiolipin over other lipids

**Figure 1:**
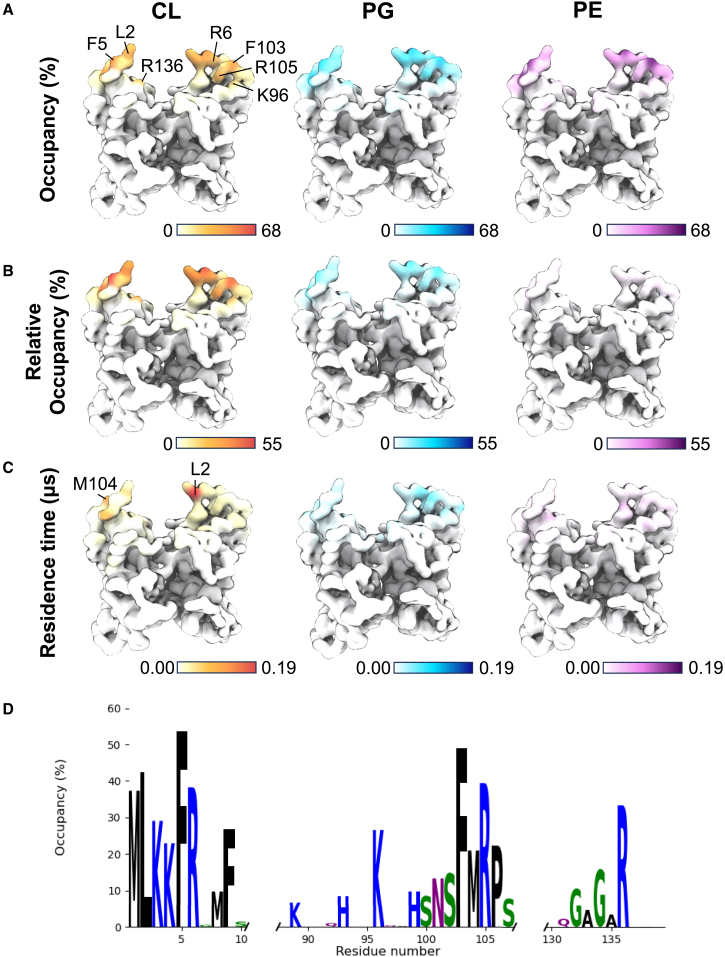
MreB dimers preferentially bind cardiolipin. (*A*) Occupancy of MreB residues with cardiolipin, PG, and PE (percentage of the simulation during which residue is interacting with a specific lipid). All color scales are 0%–68%. Residues with highest cardiolipin occupancy are labeled (note that this is a homodimer, but residues are only labeled on the monomer where the residue is most visible). (*B*) Relative occupancy of MreB residues with cardiolipin, PG, and PE, where occupancy data have been normalized to concentration of lipid in the membrane (1 CL:2 PG:7 PE). Color scale values are calculated by Occupancy divided by number of lipid molecules per cardiolipin molecule (one for cardiolipin, two for PG, seven for PE). (*C*) Residence time of MreB residues with cardiolipin, PG, and PE (average time per interaction). All color scales are 0.00 to 0.19 μs. Residues with the longest cardiolipin residence time are labeled. (*D*) Occupancy of MreB dimer residues with cardiolipin (same data as Fig. 1*A*, left), shown as a weblogo. Residues are colored by chemical properties: hydrophobic amino acids are black, basic are blue, acidic are red, and polar are green.

Initially, MD simulations were set up of MreB monomers or dimers placed in solution at a random orientation, 10 nm away from a model *E. coli* membrane (70% PE, 20% PG, and 10% CL) (23). In all simulations, MreB rotated freely in solution before spontaneously binding to the membrane. These simulations showed a consistent orientation of MreB binding to the membrane, with the N-terminal helix and surrounding residues binding the membrane. The PyLipID (27) toolkit was used to analyze interactions between MreB residues and each of the lipids in the membrane. Specifically, occupancy and residence time were used to assess interactions, where an interaction begins when beads get less than 0.475 nm apart and ends when they are more than 0.8 nm apart, to allow for fluctuation in coarse-grained bead position. Occupancy reports the percentage of the simulation time in which a specific lipid type is interacting with a specific residue, while residence time indicates how long each interaction with a specific lipid molecule lasts. And in the case of occupancy, relative occupancy was also calculated to account for the different concentrations of each lipid type in the membrane (occupancy value divided by 7 for PE and by 2 for PG). This analysis showed a preference for MreB binding to CL-individual interactions (residence time) were longer, and relative occupancy of CL was much greater than the other lipids (Figs. 1 and S3).

The residues from MreB dimers that interact most with CL are F5, F103, L2, M104, R6, K96, R105, and R136. F5, F103, and L2 are hydrophobic residues and interact more with the tail of CL than with the headgroup, as would be expected due to hydrophobic interactions (Fig. S4). M104, however, is also hydrophobic and interacts at a similar level with each part of CL, although slightly more with the headgroup. R6, K96, R105, and R136 are basic residues, and we observe that these residues, along with other arginine and lysine residues, primarily interact with the negatively charged headgroup of CL. These basic residues of MreB are therefore hypothesized to provide specificity for CL due to the electrostatic interactions formed with the headgroup—to be tested later in this paper.

An AlphaFold 3 prediction of MreB with CL shows CL binding around the residues that are identified from this interaction analysis (Fig. S5).

### MreB filaments recruit cardiolipin and cause membrane bending

**Figure 2:**
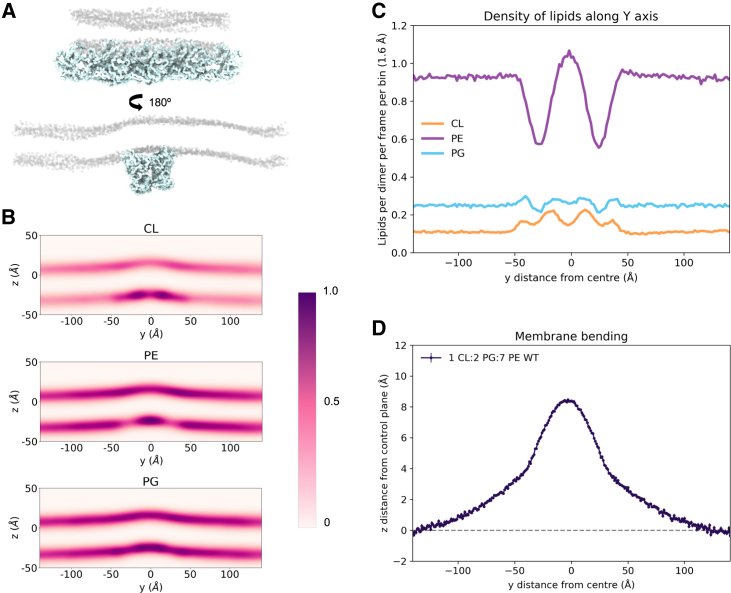
MreB filaments recruit cardiolipin, displace PE, and cause membrane bending. (*A*) A snapshot of the simulation of MreB filament (*cyan*) with a lipid bilayer (*phosphate beads shown in gray*), on the x/z plane (*upper*) and y/z plane (*lower*). (*B*) Density maps of cardiolipin, PG, and PE on the y/z plane over the course of five repeats of 5-μs simulations. MreB filaments are centered in the calculation of the density, represented by (*A*). (*C*) Density of each of the lipids in the lower leaflet across the y axis. (*D*) A plot of z-position of inner leaflet lipids against the y axis over the course of the simulation, which shows membrane bending. Standard error at each y position is plotted as vertical lines. The gray dotted line represents the average z-position at the points furthest away from the filaments—the control plane.

In most rod-shaped bacteria, MreB forms filaments that bind to the membrane and other proteins to coordinate peptidoglycan synthesis (1,2,3,4,5). Therefore, continuous MreB filaments were set up in simulations, with an MreB octamer attached by an elastic network across the periodic boundary conditions (Fig. 2 *A*). An MreB octamer was chosen based on simulations with varied MreB lengths, where an octamer was the shortest filament that had the same membrane bending effect as any longer filaments (Fig. S6). Over the course of the simulation, the average density of CL in the leaflet of the membrane that MreB binds to was approximately double where MreB was bound compared with the rest of the leaflet (Fig. 2 *B* and *C*). This recruitment of CL to the site of MreB filament binding aligns with the observation that MreB preferentially binds to CL as seen in the dimer simulations. We also observe displacement of PE from around the N-terminal helix of MreB (Fig. 2 *B* and *C*). PG density is fairly uniform compared with PE and CL, although it is also impacted by MreB binding.

In addition to CL recruitment, localized bending of the membrane was observed, perpendicular to the length of the filament (Fig. 2 *B* and *D*). The membrane appears to bend toward the outside of the cell, forming a ridge above the MreB filament. We hypothesized that this may be related to the CL recruitment, as CL is a cone-shaped lipid, so it could cause this deformation of the membrane (30,31). Quantification of the bending places this ridge at around 9 Å above the rest of the membrane (Fig. 2 *D*). Note that this localized bending is distinct from the larger scale membrane curvature and MreB filament curvature observed previously (6,32).

### MreB mutants have varied cardiolipin recruitment and bending

**Figure 3:**
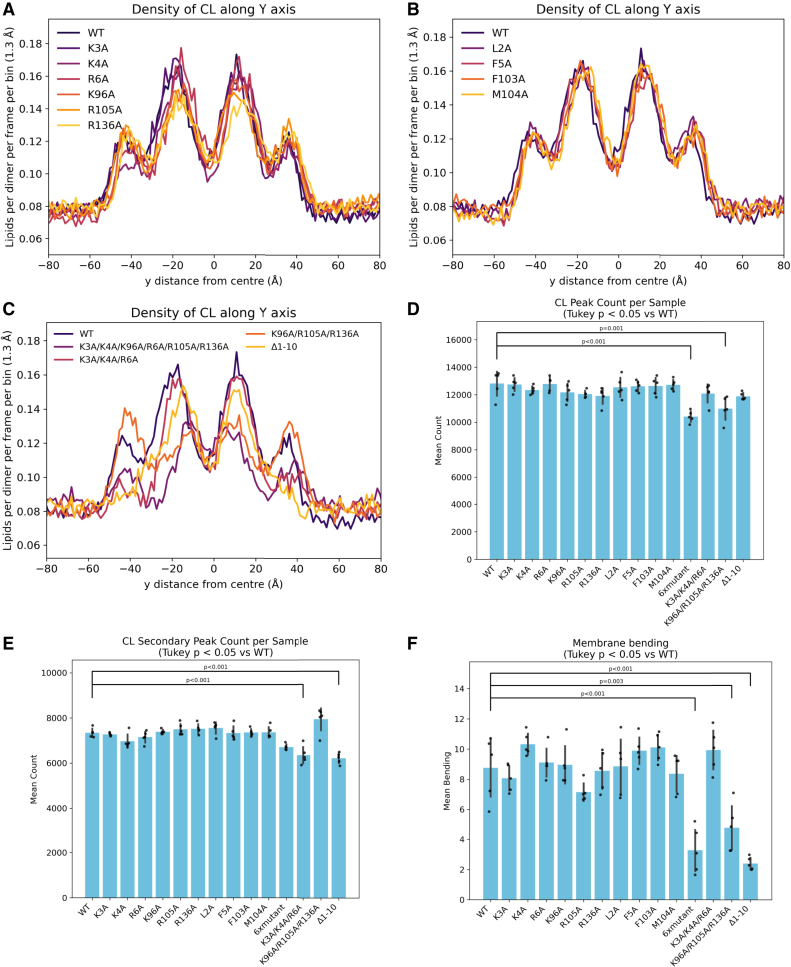
Cardiolipin density and membrane bending with MreB mutants. (*A–C*) Cardiolipin density in the lower leaflet of the membrane across the y axis, in simulations with MreB filaments (*centered*) containing different mutations. Mutations are either of basic residues (*A*), hydrophobic residues (*B*), or multiple residues including removal of residues 1–10 (*C*). The plotted y axis is shortened here compared with the simulation box, to focus on the region where MreB is bound to the membrane. (*D* and *E*) Lipid enrichment measure at central peaks (*D*) or secondary peaks (*E*) on plots (*A*)–(*C*), with significant differences between WT and other conditions labeled (from ANOVA and Tukey post hoc test). (*F*) Membrane bending by MreB containing different mutations. Bending is quantified by calculating the difference between the maximum average z-position on the y axis and the control z plane (average z-position at the points furthest away from the filaments). Significant differences between WT and other conditions are labeled (from ANOVA and Tukey post hoc test). Error bars indicate standard deviation.

MreB residues from dimers that were shown to interact highly with CL were mutated to alanine in individual point mutations R6A, K96A, R105A, and R136A (basic residues, Fig. 3 *A*) and L2A, F5A, F103A, and M104A (hydrophobic residues, Fig. 3 *B*).

All the single mutants showed a statistically similar level of CL recruitment and membrane bending to the WT (*p* > 0.05) (Fig. 3). This implies a level of redundancy in the residues required for CL recruitment and membrane bending. We therefore investigated further mutants where multiple residues were mutated at once, since single-point mutations appear to have little impact. Given the anionic headgroup of CL, and the potential role of CL recruitment in membrane bending, clusters of basic residues were chosen for these multiple mutations. Basic residues K3, K4, and R6 are all located on the N-terminal helix, which when removed dramatically decreases bending (see later in this section). Residues K96, R105, and R136 are all located on top of MreB, close in space, and were found to interact with CL in the previous analysis. Therefore, these two clusters of basic residues were all mutated to alanine, either one cluster (K3A/K4A/R6A), the other cluster (K96A/R105A/R136A), or both clusters (K3A/K4A/R6A/K96A/R105A/R136A, or “6× mutant”). The K3A/K4A/R6A mutant had significantly reduced CL recruitment at the secondary peaks of CL density (*p* < 0.001) (Fig. 3 *C* and *E*). However, membrane bending was not significantly decreased by these mutations (Fig. 3 *F*). The K96A/R105A/R136A mutant, however, showed significantly decreased CL recruitment at the larger CL density peaks (*p* = 0.001) (Fig. 3 *C* and *D*) and decreased bending to almost half that was caused by the wild-type filaments (*p* = 0.003) (Fig. 3 *F*). The K3A/K4A/R6A/K96A/R105A/R136A mutant had significantly decreased CL recruitment at the larger CL peak (*p* < 0.001) (Fig. 3 *D*), slightly but not significantly decreased CL recruitment at the secondary peak (Fig. 3 *E*), and combined, reduced bending even more than the K96A/R105A/R136A mutant (*p* < 0.001) (Fig. 3 *F*). This confirms that although there is redundancy in residues that recruit CL, these residues collectively play a key role in CL recruitment and membrane bending.

**Figure 4:**
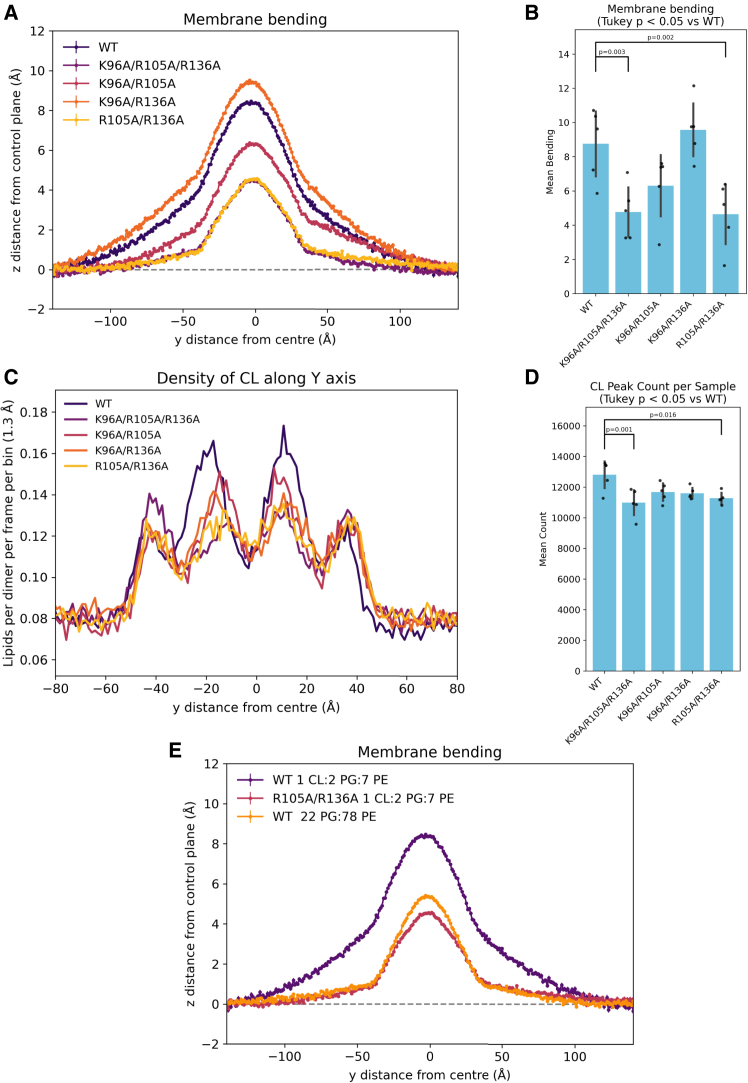
Cardiolipin density and membrane bending with MreB double and triple mutants. (*A*) Membrane bending of MreB double mutants, compared with WT and triple mutant K96A/R105A/R136A. (*B*) Quantification of bending in (*A*), with significant differences between WT and other conditions labeled (from ANOVA and Tukey post hoc test). Error bars indicate standard deviation. (*C*) Cardiolipin density of MreB double mutants, compared with WT and triple mutant K96A/R105A/R136A. (*D*) Lipid enrichment measure at central peaks on plot (*C*), with significant differences between WT and other conditions labeled (from ANOVA and Tukey post hoc test). Error bars indicate standard deviation. (*E*) Membrane bending by WT MreB or R105A/R136A of a 1 CL:2 PG:7 PE membrane, compared with WT MreB of a 22 PG:78 PE membrane.

Since we had tested both single and triple mutants, and the triple mutant K96A/R105A/R136A affected bending, we also tested double mutants from these three residues. Double mutant R105A/R136A had almost identical membrane bending to the K96A/R105A/R136A mutants (*p* = 1.000), implying that these two residues—R105 and R136—are important for bending (Fig. 4 *A* and *B*). CL recruitment was also similar in the K96A/R105A/R136A and R105A/R136A mutants (*p* = 1.000 at CL peak, *p* = 0.562 at CL secondary peak) (Figs. 4 *C*, *D*, and S8). CL interactions with residues R105 and R136 are not replaced by interactions with other residues when these residues are mutated to alanine (Fig. S12). When this double mutant is compared to WT MreB filaments with a 22 PG:78 PE membrane, the shape of membrane bending is extremely similar (*p* = 1.000) (Fig. 4 *E*). This indicates that the mutation of residues R105 and R136 to alanine is comparable to removing CL from the membrane, suggesting that their role in bending is directly related to their recruitment of CL.

**Figure 5:**
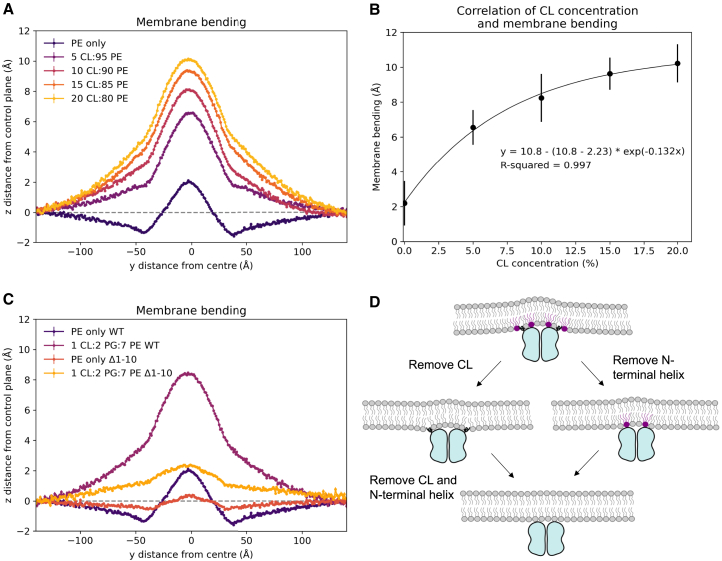
Membrane composition affects membrane bending. (*A*) Bending of membrane by WT MreB filaments with varied cardiolipin concentrations in the membrane. (*B*) Correlation of CL concentration and membrane bending. Bending from plot (*A*) is quantified by calculating the difference between the maximum average z-position on the y axis and the control z plane (average z-position at the points furthest away from the filaments). Error bars indicate standard deviation. (*C*) Membrane bending with either 1 CL:2 PG:7 PE or PE-only membrane, and with either WT MreB or MreB missing the N-terminal helix (Δ1-10). Bending is plotted as average z-position of phosphate bead of lipids in the inner leaflet at each y position. Standard error at each y position is plotted as vertical lines. The gray dotted line represents the average z-position at the points furthest away from the filaments—the control plane. (*D*) A schematic of membrane bending by MreB and the changes that removing CL and/or the N-terminal helix have on bending. Each image relates to a line from plot (*B*): 1 CL:2 PG:7 PE WT = left; PE-only WT = upper center, 1 CL:2 PG:7 PE Δ1-10 = lower center; PE-only Δ1-10 = right.

An additional mutant was tested, where the N-terminal helix (residues 1–10) was completely removed; this mutant is referred to as Δ1-10. This N-terminal amphipathic helix appears to have many interactions with the membrane and sticks into the membrane slightly, so it not only interacts with lipid headgroups but also the tails. The Δ1-10 mutant recruited CL to a similar extent at the main CL peak (Fig. 3 *C* and *D*), but to a significantly lesser extent at the secondary CL peak where the N-terminal helix would interact with the membrane (*p* < 0.001) (Fig. 3 *C* and *E*). There is also significantly less PE displacement with Δ1-10 (*p* < 0.001) (Fig. S9 *B*), and membrane bending is significantly reduced (*p* < 0.001) (Fig. 3 *F*), suggesting that the N-terminal helix of *E. coli* MreB forms interactions with the membrane that are important for membrane bending. Although some bending is still observed, the distinctive kink in the membrane around 40 Å from the center of the filament is lost (Fig. 5 *C*). This implies that the N-terminal helix is responsible for this kink in the membrane, likely related to displacement of PE and other lipids.

### Lipid concentration impacts membrane bending

So far, our observations point to a potential role of CL recruitment in the bending of membranes. To further investigate this, the effect of altering lipid concentrations on membrane bending was tested. Another aim here was to observe if PE displacement was due solely to CL replacing it, or if MreB actively displaces PE even without CL being present.

Simulations of MreB filaments with membranes containing PE and varied concentrations of CL (0%, 5%, 10%, 15%, 20%) showed that increasing CL concentration does result in increased membrane bending (Fig. 5 *A*). This increase is less between 15% and 20% CL, suggesting that MreB is becoming saturated with CL around this concentration. This is further supported by the similar increase in CL density at the MreB binding site in membrane with 15% or 20% CL (Fig. S14).

Although not to the same extent as CL, PG density is also overall increased at MreB binding sites compared with the rest of the same leaflet (Fig. 2 *C*). This is likely due to its anionic headgroup interacting with basic residues on MreB, similar to CL (Fig. S13). To investigate the role of PG recruitment, simulations were run with only PE and PG membranes, with varied PG concentration (0%, 5%, 10%, 15%, 20%). These results showed a slight increase in bending with increasing PG concentration up to 10% PG (Fig. S15 *A*). However, the bending was less pronounced than in membranes containing CL.

The increase in bending caused by increasing CL concentration can be explained by the cone shape of CL, which would cause distortion of the membrane (30,31). This is validated by a simulation where CL is replaced with a neutral CL, and similar bending is observed in comparison with charged CL (Fig. S16). PG, however, is almost cylindrical, so it is difficult to elucidate a mechanism by which PG recruitment results in membrane bending (33,34). DOPG has very slight curvature in the presence of divalent cations, and although divalent cations are not present in MreB, PG does interact with basic residues, so potentially, there is slight curvature caused by PG recruitment (33,34). Both CL and PG have negatively charged headgroups, so this accumulation of charged headgroups may also play a role in bending. Alternatively, an overall modification in lipid density at the filament binding site may result in buckling of the membrane for a more thermodynamically favorable state. PG is recruited to a greater extent when CL is absent, to the same residues as CL, so potentially, there is a preference for binding CL, but PG can replace these interactions in the absence of CL (Fig. S13).

In a membrane lacking CL, MreB filaments are still capable of bending the membrane, although to a lesser extent than in membranes with CL. This suggests that although CL recruitment has a role in membrane bending, it is not the only way that MreB causes bending. PE is displaced even in PE-only membranes, indicating that the displacement is by MreB directly, not by the recruitment of CL that then displaces PE.

CL is also still recruited to MreB binding sites where the basic residues have been mutated, although to a significantly lesser extent (Fig. 4 *C* and *D*). This may be due to CL’s affinity for negatively curved regions of membranes, where in this case the curvature is caused by the N-terminal helix of MreB (35).

Membrane-only controls were performed with different CL concentrations, the remaining lipids being PE and PG. A PE-only membrane was also included. In none of these simulations lacking MreB did the membrane bend, confirming that membrane bending is a MreB-mediated effect (Fig. S15 *B*).

### MreB causes bending through two distinct mechanisms

Given that we observed decreased bending with unique bending profiles in both MreB filament systems with a PE-only membrane and simulations with the N-terminal helix of MreB removed, we hypothesized that both CL recruitment and membrane distortion by the N-terminal helix had distinct roles in membrane bending. Therefore, simulations were set up with MreB filaments with the N-terminal helix (residues 1–10) removed, in a system with a PE-only membrane, to see if the combined effect of these changes was greater than the individual modifications to the simulations. The results of these simulations show that membrane bending is almost entirely removed (Fig. 5 *C*). This implies that CL recruitment and distortion of the membrane by the N-terminal helix of MreB both play important roles in membrane bending—visualized in a schematic (Fig. 5 *D*).

It was somewhat surprising that MreB bound the membrane at all in simulations lacking the N-terminal helix of MreB and with PE-only membranes. However, it is worth noting that although MreB is not bound to the membrane in the setup of simulations with filaments, it is very close to the membrane, so residues can very rapidly form interactions with the membrane. Although many interactions are lost in this PE-only, Δ1-10 simulation, the MreB filament still has residues that can interact with the membrane, and the filament can form many interactions due to the repeated MreB units. In simulation of a Δ1-10 MreB dimer associating to a PE-only membrane, the dimer does not bind in the “correct” orientation to the membrane, and the dimer does not stay bound for the remainder of the simulation once it is bound, as occurs with WT MreB and a 1 CL:2 PG:7 PE membrane (Fig. S17). Although this means that potentially the interaction of the Δ1-10 MreB filament with a PE-only membrane is somewhat forced by the setup of the simulation, the flattening of membrane bending is an interesting result to compare to the other filament simulations.

### Interactions of MreB with lipid II

So far in this paper, we have focused on interactions of MreB with the lipids that are in highest abundance in the *E. coli* inner membrane. However, there are many other lipids in lower abundance in the membrane. Lipid II is a peptidoglycan precursor, which is flipped to the outer leaflet of the inner membrane by a lipid II flippase, to act as a substrate for the transglycosylation step of peptidoglycan synthesis. The identity of this lipid II flippase is disputed, but there is evidence for lipid II flipping by MurJ, AmJ, and/or FtsW (36,37,38,39). RodA has also been suggested as a potential flippase due to its homology to FtsW (39). Therefore, there is a potential that MreB would interact with lipid II as part of its role in the elongasome. In fact, lipid II has been observed to have a role in localization of PBPs in cell division, so if the same is true in cell elongation, then the recruitment of lipid II by MreB could be the mechanism by which MreB recruits PBPs to form the elongasome (40,41).

**Figure 6:**
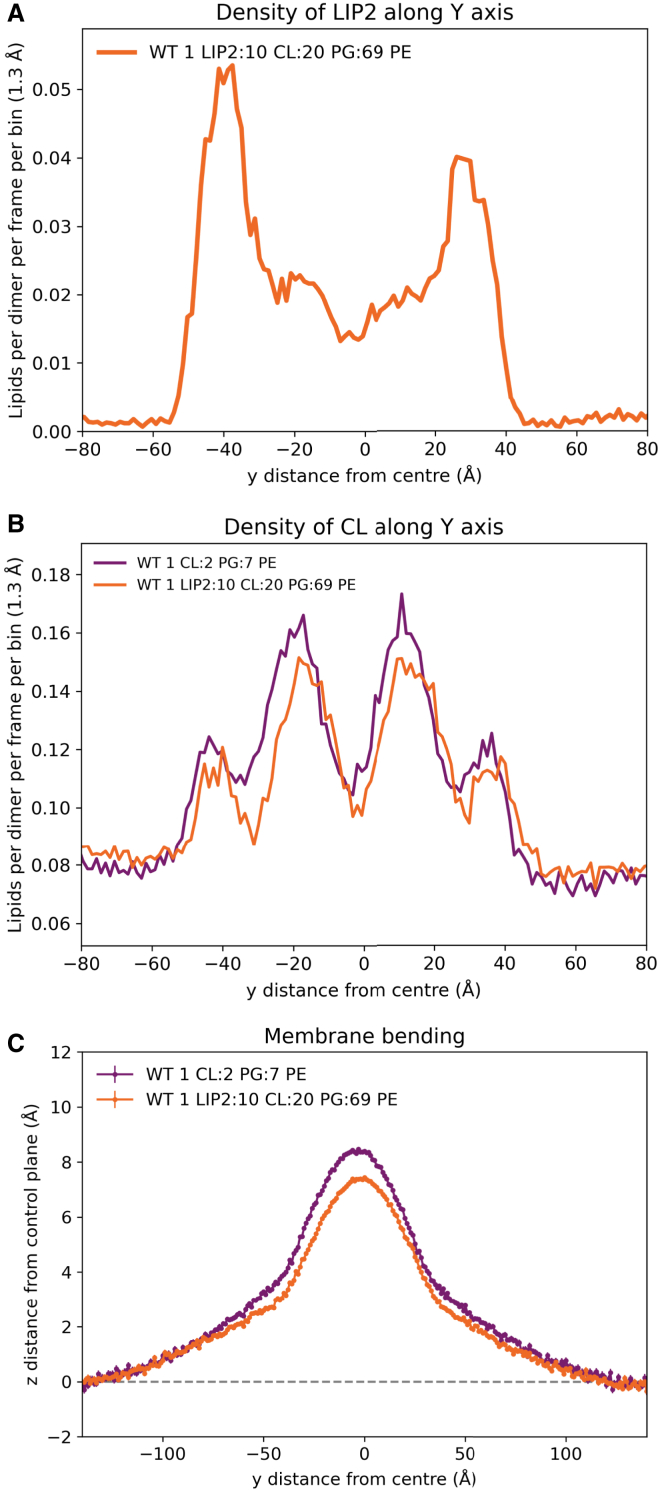
Interaction of lipid II with MreB filaments. (*A*) Lipid II (LIP2) density in the lower leaflet of the membrane across the y axis, in simulations with MreB filaments (*centered*). (*B*) CL density in the lower leaflet of the membrane across the y axis, in simulations with MreB filaments (*centered*), with membranes with or without lipid II. (*C*) Bending of membranes either with or without lipid II by WT MreB filaments.

We set up simulations of a membrane containing 1% lipid II (with 69% PE, 20% PG, 10% CL), with a continuous MreB filament. Lipid II was recruited to MreB filaments, primarily at the N-terminal helix (Figs. 6 *A* and S18 *A*). This aligns with experimental data that lipid II is involved in recruitment of MreB to the bacterial cell membrane (42). Lipid II replaces a small amount of CL around MreB and leads to a small reduction in bending (Fig. 6). However, neither the CL enrichment or membrane bending are significantly different from the system with 1 CL:2 PG:7 PE (Figs. S8 and S11).

### MD simulations of the elongasome

**Figure 7:**
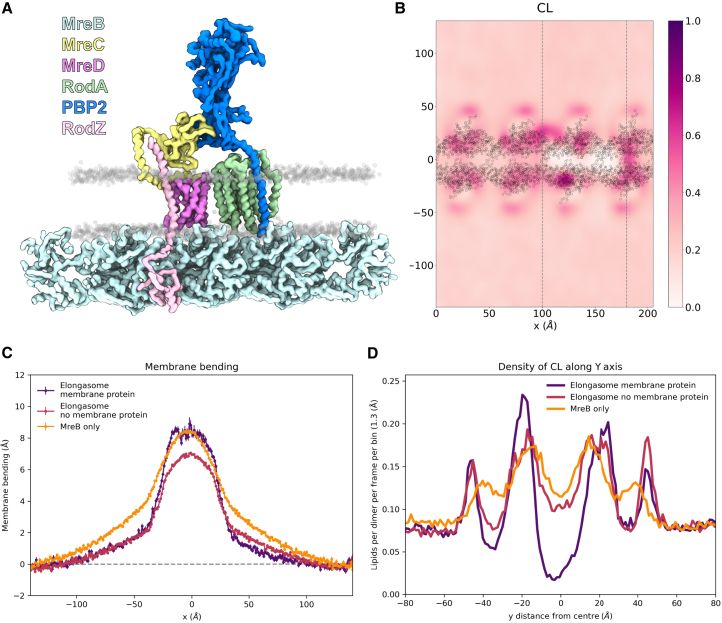
Cardiolipin recruitment and membrane bending occur when elongasome proteins are bound to MreB filaments. (*A*) The elongasome protein complex on continuous MreB filaments. Here, the protein backbones are shown in different colors, and the phosphate groups of the lipids are shown in gray. (*B*) 2D cardiolipin density (*color scale*) overlayed with the MreB backbone represented by gray circles. The vertical gray dashed lines represent what are considered the boundaries of “Elongasome membrane protein” in plots (*C*) and (*D*). (*C*) Membrane bending, plotted as average z-position of phosphate bead of lipids in the inner leaflet at each y position. (*D*) 1D cardiolipin density along the y axis of the simulation box. “Elongasome membrane protein” contains data from the elongasome simulation between the vertical gray dashed lines in (*B*), which is where the integral membrane proteins in the elongasome are situated and therefore lipid density is lower. “Elongasome no membrane protein” contains the data outside of the vertical gray dashed lines from the same simulation. “MreB only” contains data from the control simulation of just WT MreB with a 1 CL:2 PG:7 PE membrane.

In vivo, MreB is not only found binding to the membrane but also to proteins in the membrane that make up the elongasome. To consider the effects this complex may have on the interactions between MreB and the membrane, simulations were set up with a model of the elongasome bound to the continuous MreB filament (Fig. 7 *A*). The elongasome model included proteins MreB, MreC, MreD, RodA, PBP2, and RodZ.

The aim of these simulations with the elongasome was to determine whether the membrane still bends when MreB is present not only by itself but as part of the elongasome. We observed that bending did still occur, as well as recruitment of CL in a similar pattern to before, perhaps even more exaggerated, suggesting that other components of the elongasome may contribute to the accumulation of CL (Fig. 7 *B*). Since the integral membrane protein components of the elongasome did not span the entire length of the filament, for analysis of CL recruitment and membrane bending, the data were analyzed in two bins on the x axis. The “Elongasome membrane protein” bin contained data in the bin on the x axis where membrane protein components of the elongasome were located, whereas “Elongasome no membrane protein” contains the remaining data. This allowed us to observe that the membrane in fact appeared to bend more in the regions containing the membrane proteins than where they are absent, within the same simulation. This may be due to the additional recruitment of CL, or it may be an impact of having the proteins within the membrane. The shape of the membrane bending is also impacted by the presence of the elongasome proteins; although the maximum distortion on the z axis is similar, the bend is sharper. The width of the area in which CL is recruited is also increased in the elongasome simulations (Fig. 7 *B*). Although MreB began the elongasome simulation identical to the MreB-only simulations, the distance between the protofilaments is marginally increased in the elongasome simulation. It is unclear whether this would be a cause of the altered bending shape, however.

PE displacement does not appear to have been much affected by the presence of the elongasome, other than the decreased density where the integral membrane proteins are (Fig. S19 *A*). The density of PG also has a similar pattern in the elongasome simulations, although there are notable localized increases in PG around the membrane proteins of the elongasome, particularly found in between RodA and MreD (Fig. S19 *B*). This may indicate a role of PG in bridging these proteins, although further investigation into this interaction was not within the remit of this paper.

Overall, these elongasome simulations reveal that MreB continues to recruit CL, displace PE, and bend the membrane in its more native environment as part of the elongasome.

## Discussion

In this work, we uncovered a novel mechanism of membrane bending by MreB. Specifically, the N-terminal helix of MreB appears to displace lipids and physically distort the membrane, whereas positively charged residues recruit CL to where MreB is binding the membrane, causing further membrane bending due to the cone shape of CL. The role of this bending is unclear, but a few options are possible. It could play a role in keeping the MreB filaments oriented where they are aligned to negative membrane curvature by making misalignment more thermodynamically unfavorable. It could help to physically recruit membrane proteins to the elongasome or have a role in the dynamics of the elongasome as it rotates around the cell, maybe by altering membrane tension. Alternatively, since the bending is toward the peptidoglycan, bringing the peptidoglycan synthesis proteins in the elongasome closer to the peptidoglycan may allow for more efficient peptidoglycan synthesis. Given that cells still grow (spherically), at least for a short time, when MreB is inhibited, there is no current evidence to suggest that this bending is required for peptidoglycan synthesis to occur. However, disruption of MreB results in deformed cells with reduced viability, so it is useful to gain a better understanding of how MreB works (9,43,44).

The observation that removal of the N-terminal helix of MreB impairs membrane bending may be related to the role of MreB in determining cell shape. A 2011 study by Salje et al. revealed the role of the N-terminal helix of *E. coli* MreB in cell shape determination (16). Here, although MreB without the N-terminal helix (EcΔNMreB) bound to membranes to some extent, MreB-negative cells overexpressing EcΔNMreB remained spherical, whereas overexpression of WT EcMreB produced rod-shaped cells (16). This N-terminal amphipathic helix is not found in all MreB homologs, although it is found in various other Gram-negative bacteria (16). The double mutant F103A/M104A was also investigated in this study and resulted in slightly fewer rod-shaped cells than WT, but point mutations of basic residues R105 and R136 have not been performed.

The recruitment of lipid II by MreB observed in simulations supports experimental data that suggest a role of lipid II in MreB association with the membrane (42,45). It has also been suggested that lipid II is what localizes PBPs during cell elongation, so potentially MreB indirectly recruits PBPs by first recruiting lipid II, which PBPs then localize to (40,41).

Currently, almost all MreB inhibitors bind in or adjacent to the nucleotide binding site, inhibiting ATPase activity and elongation of MreB filaments (46). However, with a better understanding of how MreB binds the membrane, inhibitors could potentially be designed to interfere with the binding of MreB to the membrane or proteins in the elongasome. For example, one antibiotic—MbiA—is predicted to bind at the interaction site between RodZ and MreB (47). Further development of antibiotics designed to inhibit such interactions may prove a valuable method in combating antimicrobial resistance.

## Conclusions

To conclude, this paper presents the novel finding that *E. coli* MreB preferentially binds to CL over lipids PE and PG in the bacterial cell membrane. CL is recruited at least in part by basic residues on MreB. This recruitment of CL, as well as membrane interactions with the partially conserved N-terminal amphipathic helix of MreB, causes a local bending of the membrane above MreB filaments toward the peptidoglycan, perpendicular to the length of the filaments. This occurs in the absence or presence of lipid II or additional elongasome proteins.

## Acknowledgments

We offer thanks to the past and present members of the Stansfeld group for their constant support. This project is funded by the 10.13039/501100000265Medical Research Council through the Warwick MRC DTP. The Stansfeld laboratory received funding from Wellcome, MRC, BBSRC, EPSRC, NIH, JPIAMR, and the Howard Dalton Centre. This work utilized ARCHER2 resources provided by the UK High-End Computing Consortium for Biomolecular Simulation (HECBioSim, http://www.hecbiosim.ac.uk), supported by EPSRC (grant EP/R029407/1). We also acknowledge the Scientific Computing Research Technology Platform (SCRTP) at the University of Warwick for computational resources. Additional computations were performed on Sulis at HPC Midlands+, funded by EPSRC (grant EP/T022108/1).

## Author contributions

B.W.A.B. and P.J.S. designed the study. B.W.A.B. performed most of the MD simulations and analysis with input from P.J.S. and T.D.H.B. A.D.Q.H. performed atomistic simulations of MreB monomer with a membrane. B.W.A.B. wrote the first draft. B.W.A.B., P.J.S., and T.D.H.B. wrote the final paper.

## Declaration of interests

The authors declare no competing interests.

## Supporting material

Document S1. Figures S1–S19

Document S2. Article plus supporting material

## References

[1] van den Ent F., Izoré T., Löwe J. (2014). Bacterial actin MreB forms antiparallel double filaments. eLife.

[2] Shi H., Bratton B.P., Huang K.C. (2018). How to Build a Bacterial Cell: MreB as the Foreman of E. coli Construction. Cell.

[3] Nguyen L.T., Gumbart J.C., Jensen G.J. (2015). Coarse-grained simulations of bacterial cell wall growth reveal that local coordination alone can be sufficient to maintain rod shape. Proc. Natl. Acad. Sci. USA.

[4] Garner E.C., Bernard R., Mitchison T. (2011). Coupled, circumferential motions of the cell wall synthesis machinery and MreB filaments in B. subtilis. Science.

[5] Domínguez-Escobar J., Chastanet A., Carballido-López R. (2011). Processive movement of MreB-associated cell wall biosynthetic complexes in bacteria. Science.

[6] Hussain S., Wivagg C.N., Garner E.C. (2018). MreB filaments align along greatest principal membrane curvature to orient cell wall synthesis. eLife.

[7] Amir A., Nelson D.R. (2012). Dislocation-mediated growth of bacterial cell walls. Proc. Natl. Acad. Sci. USA.

[8] White C.L., Kitich A., Gober J.W. (2010). Positioning cell wall synthetic complexes by the bacterial morphogenetic proteins MreB and MreD. Mol. Microbiol..

[9] Kruse T., Bork-Jensen J., Gerdes K. (2005). The morphogenetic MreBCD proteins of Escherichia coli form an essential membrane-bound complex. Mol. Microbiol..

[10] Rice L.B. (2008). Federal Funding for the Study of Antimicrobial Resistance in Nosocomial Pathogens: No ESKAPE. J. Infect. Dis..

[11] Bryan E.J., Sagong H.Y., Pilch D.S. (2022). TXH11106: A Third-Generation MreB Inhibitor with Enhanced Activity against a Broad Range of Gram-Negative Bacterial Pathogens. Antibiotics (Basel).

[12] Hett E.C., Rubin E.J. (2008). Bacterial growth and cell division: a mycobacterial perspective. Microbiol. Mol. Biol. Rev..

[13] Kieser K.J., Rubin E.J. (2014). How sisters grow apart: mycobacterial growth and division. Nat. Rev. Microbiol..

[14] Nygaard R., Graham C.L.B., Mancia F. (2023). Structural basis of peptidoglycan synthesis by E. coli RodA-PBP2 complex. Nat. Commun..

[15] Sjodt M., Rohs P.D.A., Kruse A.C. (2020). Structural coordination of polymerization and crosslinking by a SEDS–bPBP peptidoglycan synthase complex. Nat. Microbiol..

[16] Salje J., van den Ent F., Löwe J. (2011). Direct Membrane Binding by Bacterial Actin MreB. Mol. Cell.

[17] Mao W., Renner L.D., Carballido-Lopez R. (2023). On the role of nucleotides and lipids in the polymerization of the actin homolog MreB from a Gram-positive bacterium. eLife.

[18] Jumper J., Evans R., Hassabis D. (2021). Highly accurate protein structure prediction with AlphaFold. Nature.

[19] Schrödinger L.D. (2022).

[20] Abramson J., Adler J., Jumper J.M. (2024). Accurate structure prediction of biomolecular interactions with AlphaFold 3. Nature.

[21] Souza P.C.T., Alessandri R., Marrink S.J. (2021). Martini 3: a general purpose force field for coarse-grained molecular dynamics. Nat. Methods.

[22] Nugent T., Jones D.T. (2013). Membrane protein orientation and refinement using a knowledge-based statistical potential. BMC Bioinf..

[23] Murzyn K., Róg T., Pasenkiewicz-Gierula M. (2005). Phosphatidylethanolamine-phosphatidylglycerol bilayer as a model of the inner bacterial membrane. Biophys. J..

[24] Wassenaar T.A., Ingólfsson H.I., Marrink S.J. (2015). Computational Lipidomics with insane: A Versatile Tool for Generating Custom Membranes for Molecular Simulations. J. Chem. Theor. Comput..

[25] Abraham M.J., Murtola T., Lindahl E. (2015). GROMACS: High performance molecular simulations through multi-level parallelism from laptops to supercomputers. SoftwareX.

[26] Kim H., Fábián B., Hummer G. (2023). Neighbor List Artifacts in Molecular Dynamics Simulations. J. Chem. Theor. Comput..

[27] Song W., Corey R.A., Sansom M.S.P. (2022). PyLipID: A Python Package for Analysis of Protein–Lipid Interactions from Molecular Dynamics Simulations. J. Chem. Theor. Comput..

[28] PLUMED consortium (2019). Promoting transparency and reproducibility in enhanced molecular simulations. Nat. Methods.

[29] Humphrey W., Dalke A., Schulten K. (1996). VMD: Visual molecular dynamics. J. Mol. Graph..

[30] Rand R.P., Sengupta S. (1972). Cardiolipin forms hexagonal structures with divalent cations. Biochim. Biophys. Acta.

[31] Powell G.L., Hui S.W. (1996). Tetraoleoylpyrophosphatidic acid: a four acyl-chain lipid which forms a hexagonal II phase with high curvature. Biophys. J..

[32] Shi H., Quint D.A., Huang K.C. (2020). Chiral twisting in a bacterial cytoskeletal polymer affects filament size and orientation. Nat. Commun..

[33] Oliver P.M., Crooks J.A., Weibel D.B. (2014). Localization of Anionic Phospholipids in Escherichia coli Cells. J. Bacteriol..

[34] Alley S.H., Ces O., Templer R.H. (2008). X-ray diffraction measurement of the monolayer spontaneous curvature of dioleoylphosphatidylglycerol. Chem. Phys. Lipids.

[35] Beltrán-Heredia E., Tsai F.C., Monroy F. (2019). Membrane curvature induces cardiolipin sorting. Commun. Biol..

[36] Sham L.-T., Butler E.K., Ruiz N. (2014). MurJ is the flippase of lipid-linked precursors for peptidoglycan biogenesis. Science.

[37] Meeske A.J., Sham L.T., Rudner D.Z. (2015). MurJ and a novel lipid II flippase are required for cell wall biogenesis in Bacillus subtilis. Proc. Natl. Acad. Sci. USA.

[38] Mohammadi T., van Dam V., Breukink E. (2011). Identification of FtsW as a transporter of lipid-linked cell wall precursors across the membrane. EMBO J..

[39] Ruiz N. (2015). Lipid Flippases for Bacterial Peptidoglycan Biosynthesis. Lipid Insights.

[40] Scheffers D.J., Pinho M.G. (2005). Bacterial cell wall synthesis: new insights from localization studies. Microbiol. Mol. Biol. Rev..

[41] Pinho M.G., Errington J. (2005). Recruitment of penicillin-binding protein PBP2 to the division site of Staphylococcus aureus is dependent on its transpeptidation substrates. Mol. Microbiol..

[42] Schirner K., Eun Y.J., Walker S. (2015). Lipid-linked cell wall precursors regulate membrane association of bacterial actin MreB. Nat. Chem. Biol..

[43] Bonez P.C., Ramos A.P., Campos M.M.A. (2016). Antibacterial, cyto and genotoxic activities of A22 compound ((S-3, 4 -dichlorobenzyl) isothiourea hydrochloride). Microb. Pathog..

[44] Iwai N., Nagai K., Wachi M. (2002). Novel S-benzylisothiourea compound that induces spherical cells in Escherichia coli probably by acting on a rod-shape-determining protein(s) other than penicillin-binding protein 2. Biosci. Biotechnol. Biochem..

[45] Scheffers D.J., Tol M.B. (2015). LipidII: Just Another Brick in the Wall?. PLoS Pathog..

[46] Awuni E. (2019). Status of Targeting MreB for the Development of Antibiotics. Front. Chem..

[47] Yakhnina A.A., Gitai Z. (2012). The small protein MbiA interacts with MreB and modulates cell shape in Caulobacter crescentus. Mol. Microbiol..

