# Supplementary material for: MreB filaments in the elongasome modulate E. coli membrane curvature
Source: Biophys J. 2025 Sep 2;124(21):3583–95. doi: 10.1016/j.bpj.2025.08.036 (PMC12709425; doi:10.1016/j.bpj.2025.08.036)
Supplement: Document S1. Figures S1–S19 [file mmc1.pdf]

Biophysical Journal, Volume *124*

Supplemental information

MreB filaments in the elongasome modulate *E. coli* membrane curvature

Becca W.A. Baileeves, Anthony D.Q. Hoang, Timothy D.H. Bugg, and Phillip J. Stansfeld

Supplementary Material

MreB Filaments in the Elongasome Modulate *E. coli* Membrane Curvature

Becca W. A. Baileeves[1,2,3], *Anthony D. Q. Hoang*[1,2], *Timothy D. H. Bugg*[3] & *Phillip J. Stansfeld*[1,3]*

[1]School of Life Sciences, University of Warwick, Coventry, CV4 7AL, UK

[2]MRC DTP, Warwick Medical School, University of Warwick, Gibbet Hill Road, Coventry, CV4 7AL, UK

[3]Department of Chemistry, University of Warwick, Coventry, CV4 7AL, UK

*to whom correspondence should be addressed:

e-mail: phillip.stansfeld@warwick.ac.uk

phone: +44 (0) 24 7652 3864

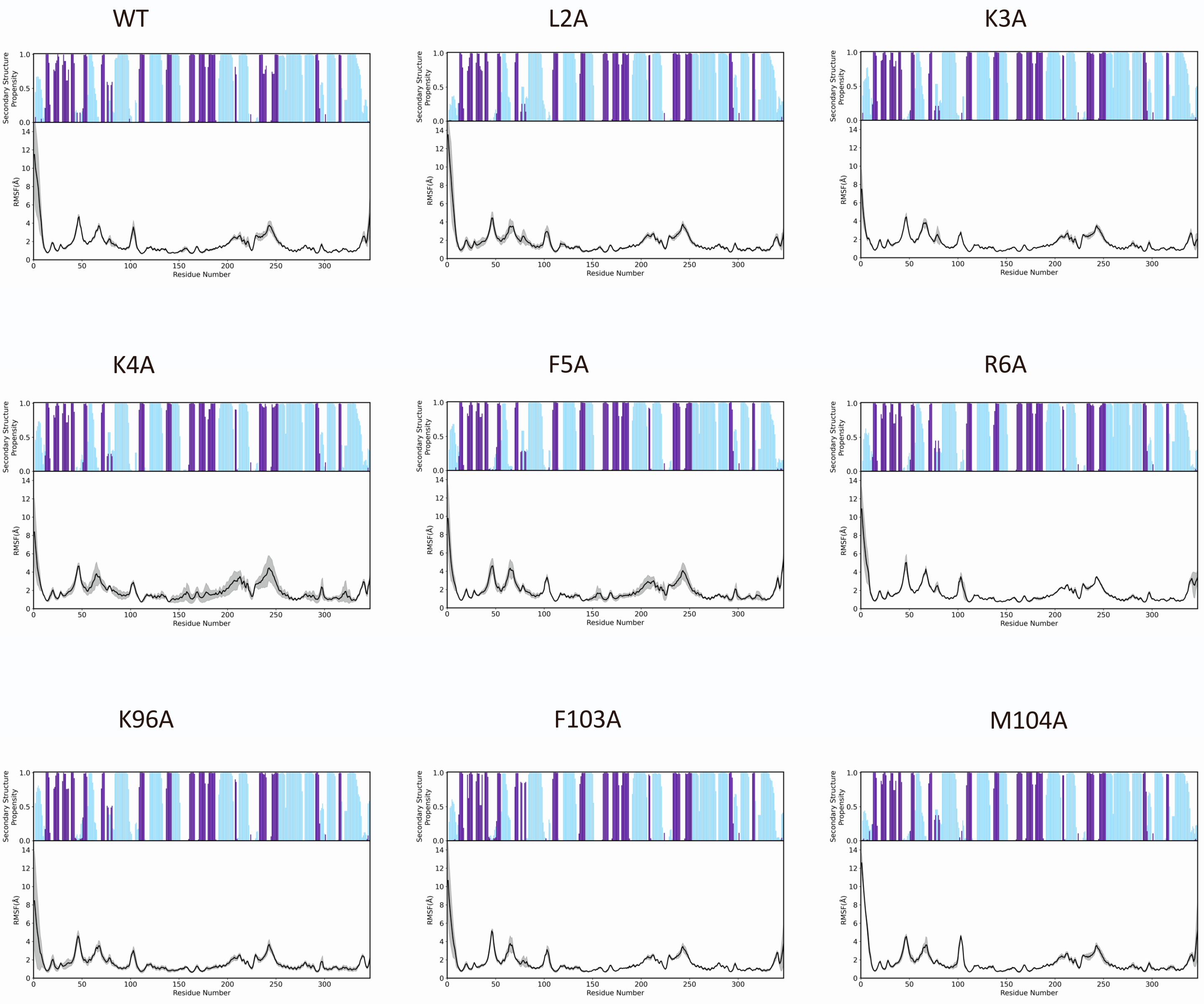

Figure S1 continued in following page.

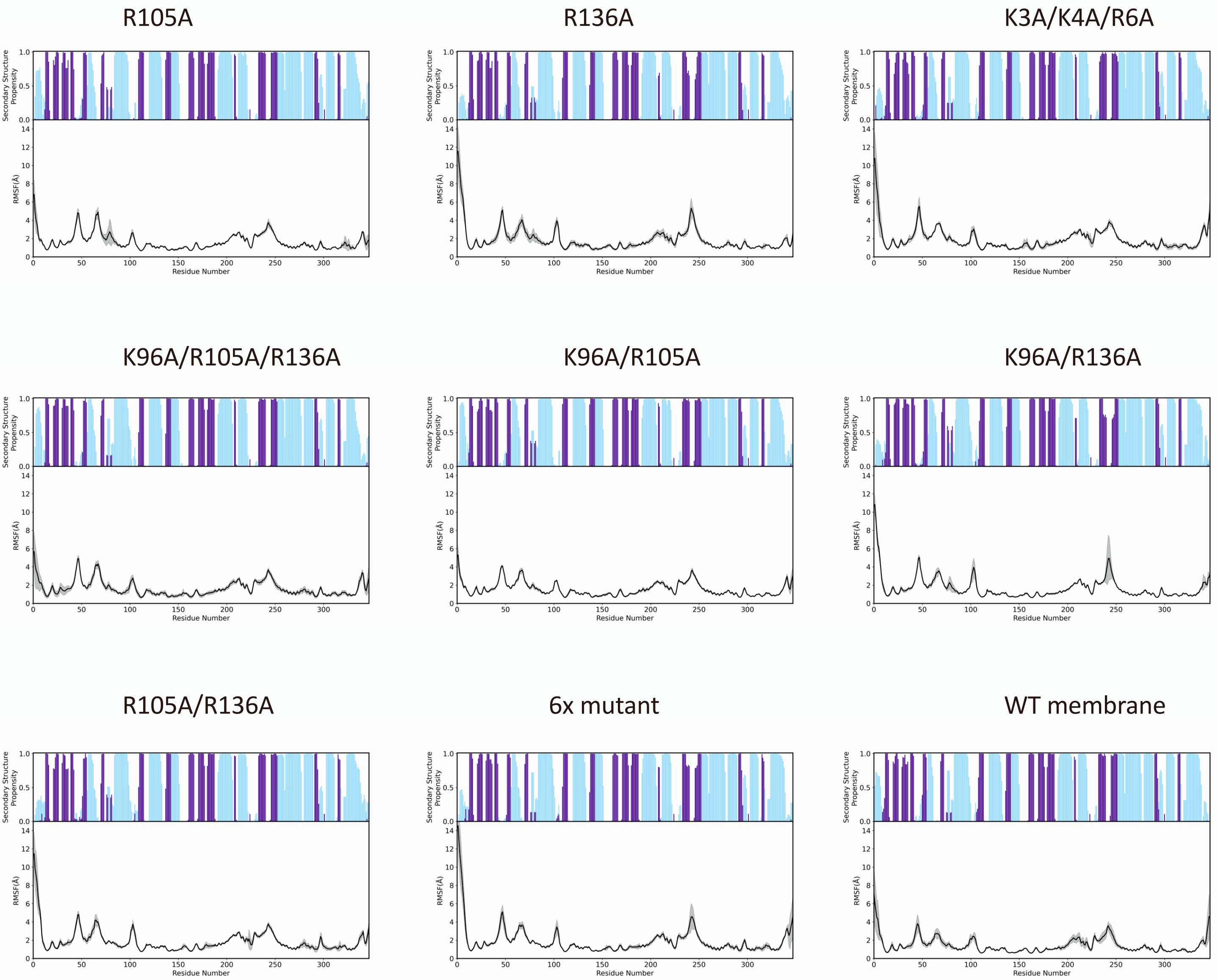

Figure S1: Secondary structure propensity and RMSF of MreB mutants in atomistic simulations. Pale blue = alpha helices, purple = beta strands. Simulations are of MreB monomers in solution apart from "WT membrane", which is a monomer peripherally bound to a 1 CL: 2 PG: 7 PE membrane.

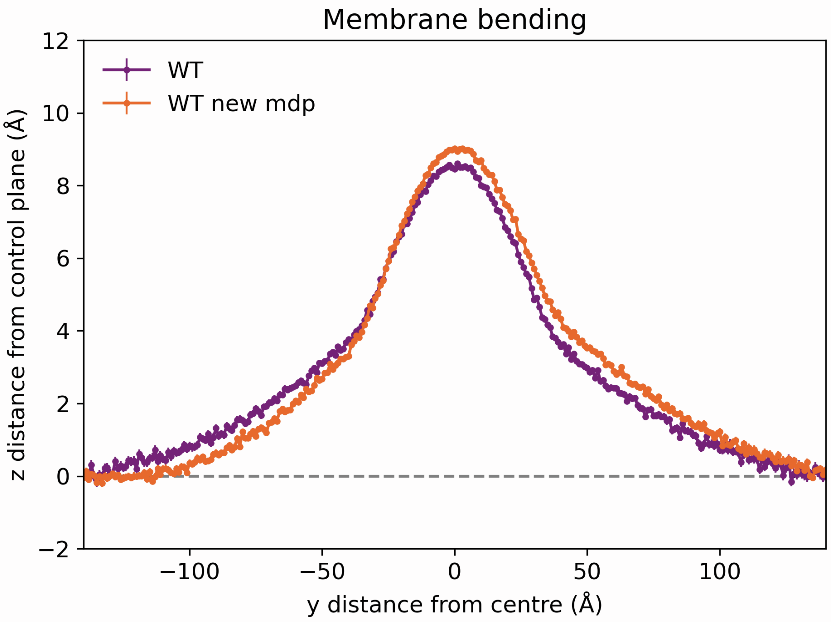

Figure S2: Membrane bending comparison with different parameters. Both systems contain an octamer of WT MreB, with a membrane of 1 CL: 2 PG: 7 PE. The "new mdp" refers to nstlist=20, verlet-buffer-tolerance=0.0002, rlist=1.35, based on a recent paper[1] as discussed in the methods section of this paper.

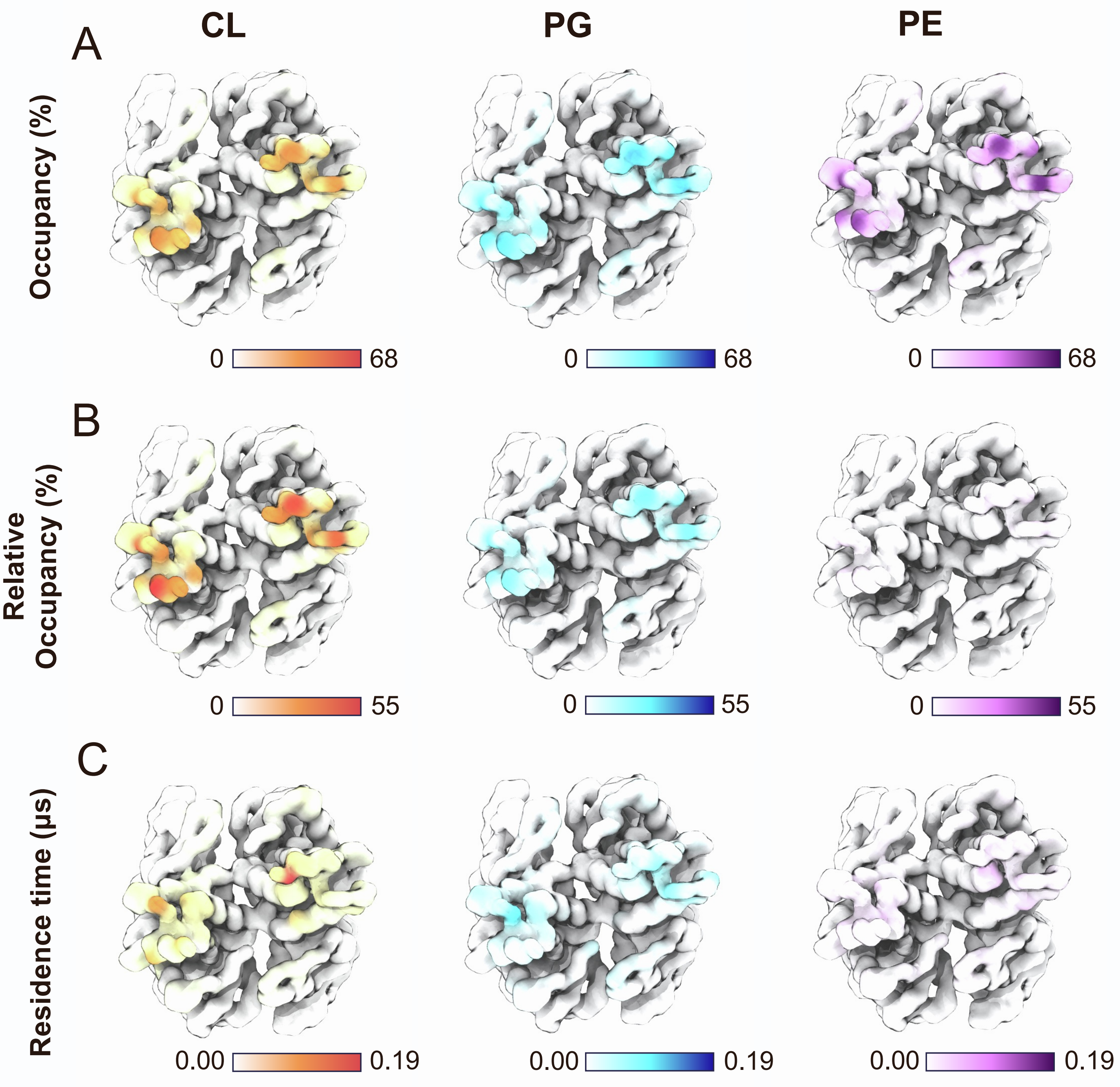

Figure S3: MreB dimer interactions with lipids, as observed from MreB-membrane interaction site ("top" of MreB). A) Occupancy of MreB residues with CL, PG and PE (percentage of the simulation during which residue is interacting with a specific lipid). All colour scales 0% to 68%. B) Relative occupancy of MreB residues with cardiolipin, PG and PE, where occupancy data has been normalized to concentration of lipid in the membrane (1 CL: 2 PG: 7 PE). Colour scales relative to each other. C) Residence time of MreB residues with cardiolipin, PG and PE (average time per interaction). All colour scales 0.00 to 0.19 µs.

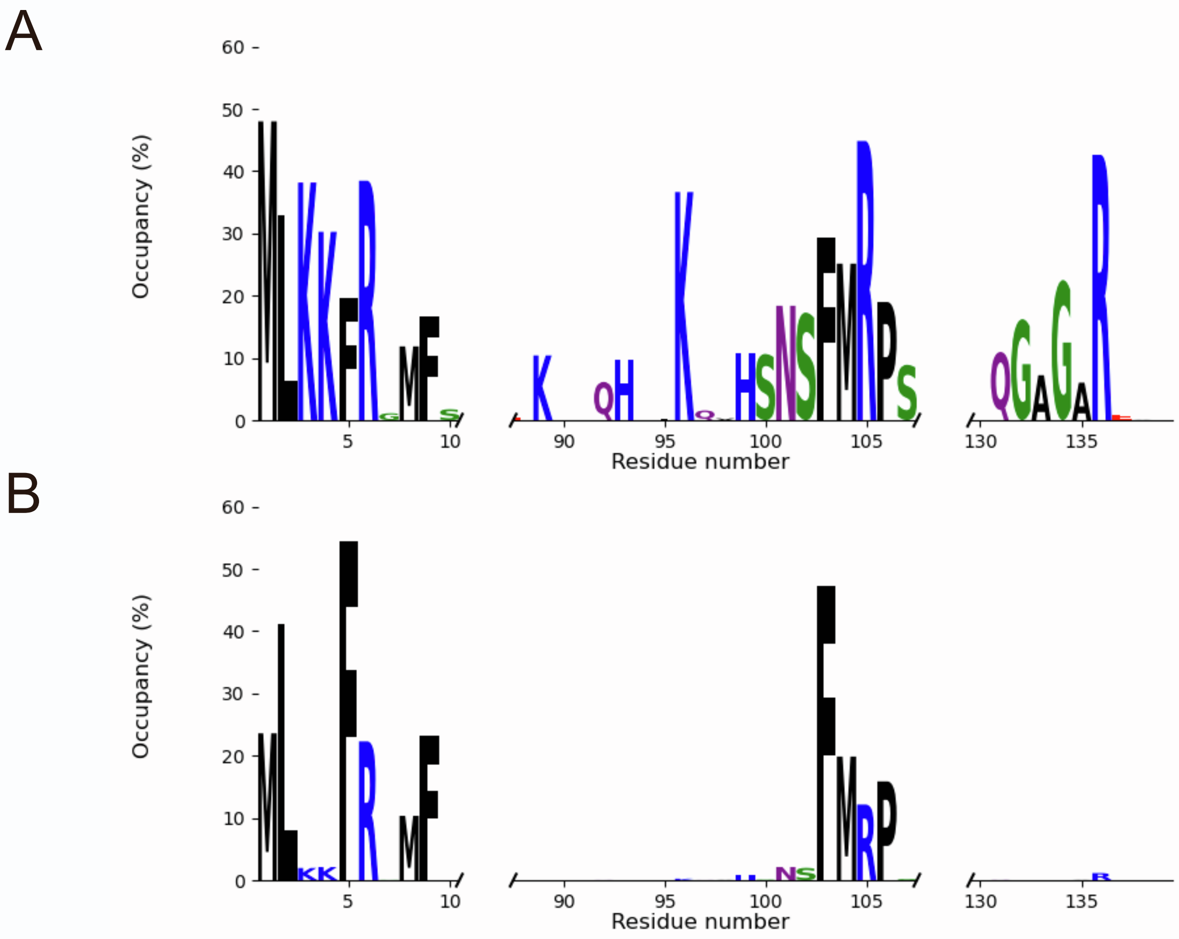

Figure S4: MreB dimer interactions with head group (A) or tail (B) of cardiolipin molecules, measured as occupancy of the relative beads within CL with MreB (percentage of the simulation during which residue is interacting with specific beads). Residues are coloured by chemical properties: hydrophobic amino acids are black, basic are blue, acidic are red, and polar are green.

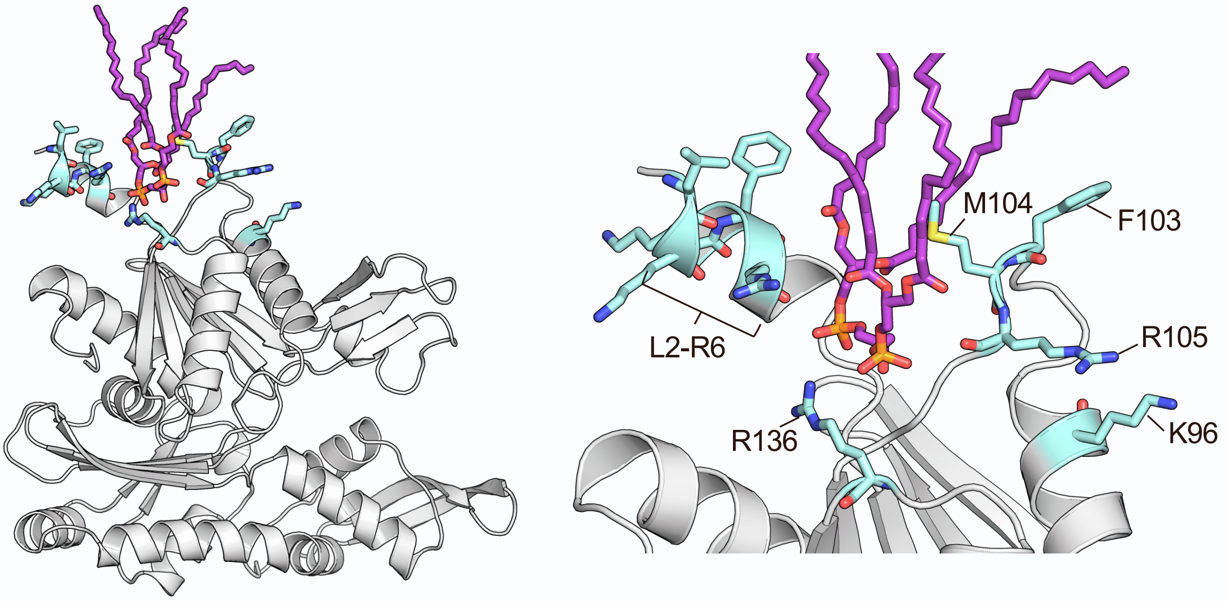

Figure S5: AlphaFold3 prediction of MreB monomer (grey/cyan) with cardiolipin (magenta). MreB residues that are shown to interact with cardiolipin in Figure 1 are shown in cyan as sticks.

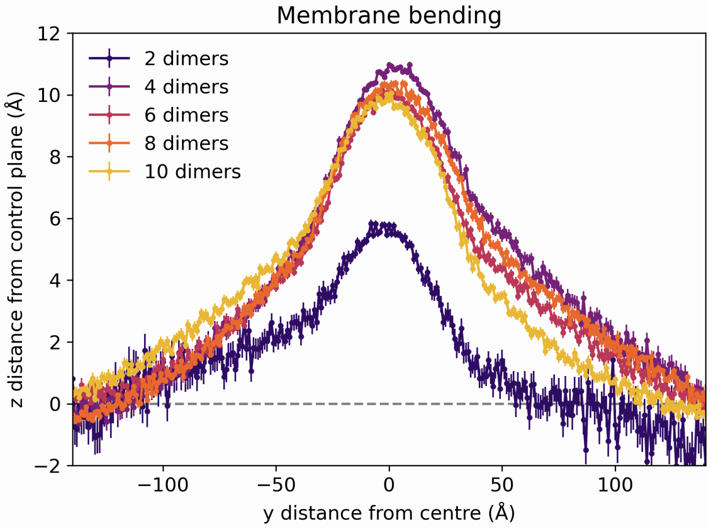

Figure S6: Bending of membrane by WT MreB filaments of different lengths. A 1 CL: 2 PG: 7 PE membrane is used. All filaments are attached across the periodic boundary by an elastic network.

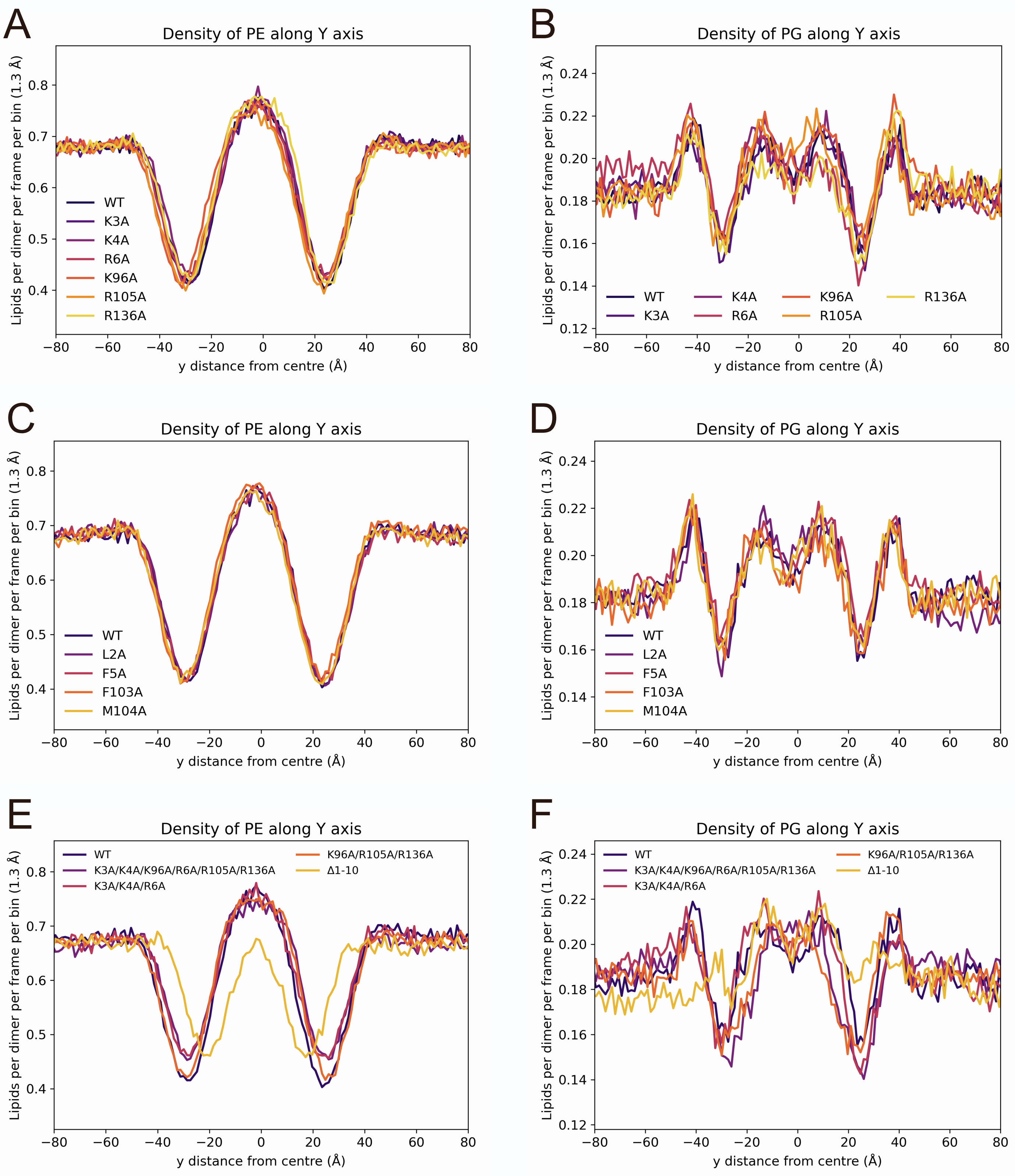

Figure S7: PE and PG density of MreB mutants plotted in Figure 3. PE (A) and PG (B) density of single mutations of basic residues. PE (C) and PG (D) density of single mutations of hydrophobic residues. PE (E) and PG (F) density of mutants with multiple mutations of basic residues, or removal of the N-terminal helix of MreB (Δ1-10).

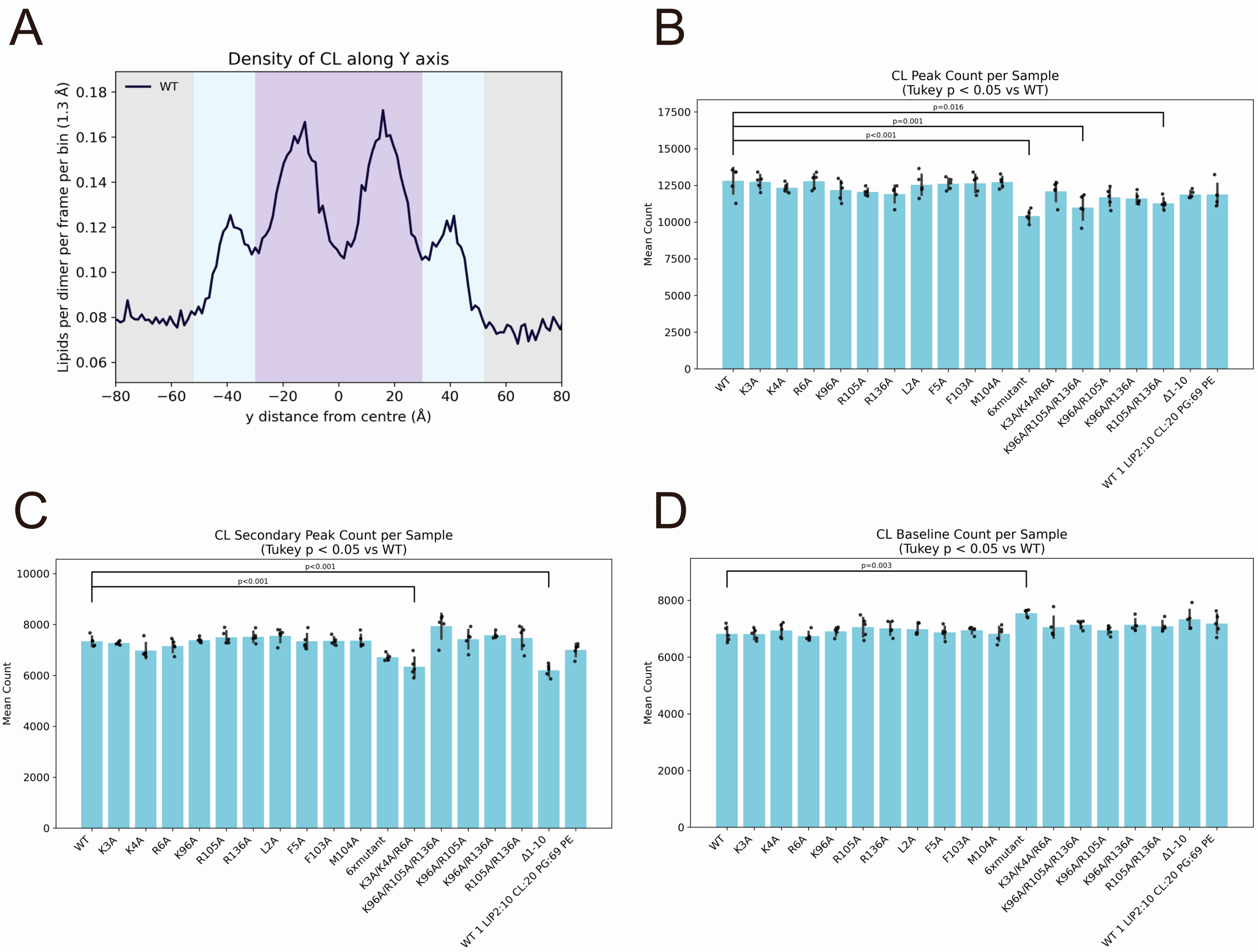

Figure S8: Cardiolipin enrichment measurements in different conditions. A) Definition of regions on the y-axis that are compared in the lipid enrichment measurements: purple = “CL Peak” (B), pale blue = “CL Secondary Peak” (C), grey = “CL Baseline” (D). Data shown in (A) is from WT MreB with 1 CL: 2 PG: 7 PE membrane. B-D) Count of cardiolipin molecules in the regions defined in (A) in different mutants and simulation conditions, all with 10% CL in the membrane. Significant differences between WT and other conditions are labelled with their p-value from an ANOVA and Tukey-HSD test.

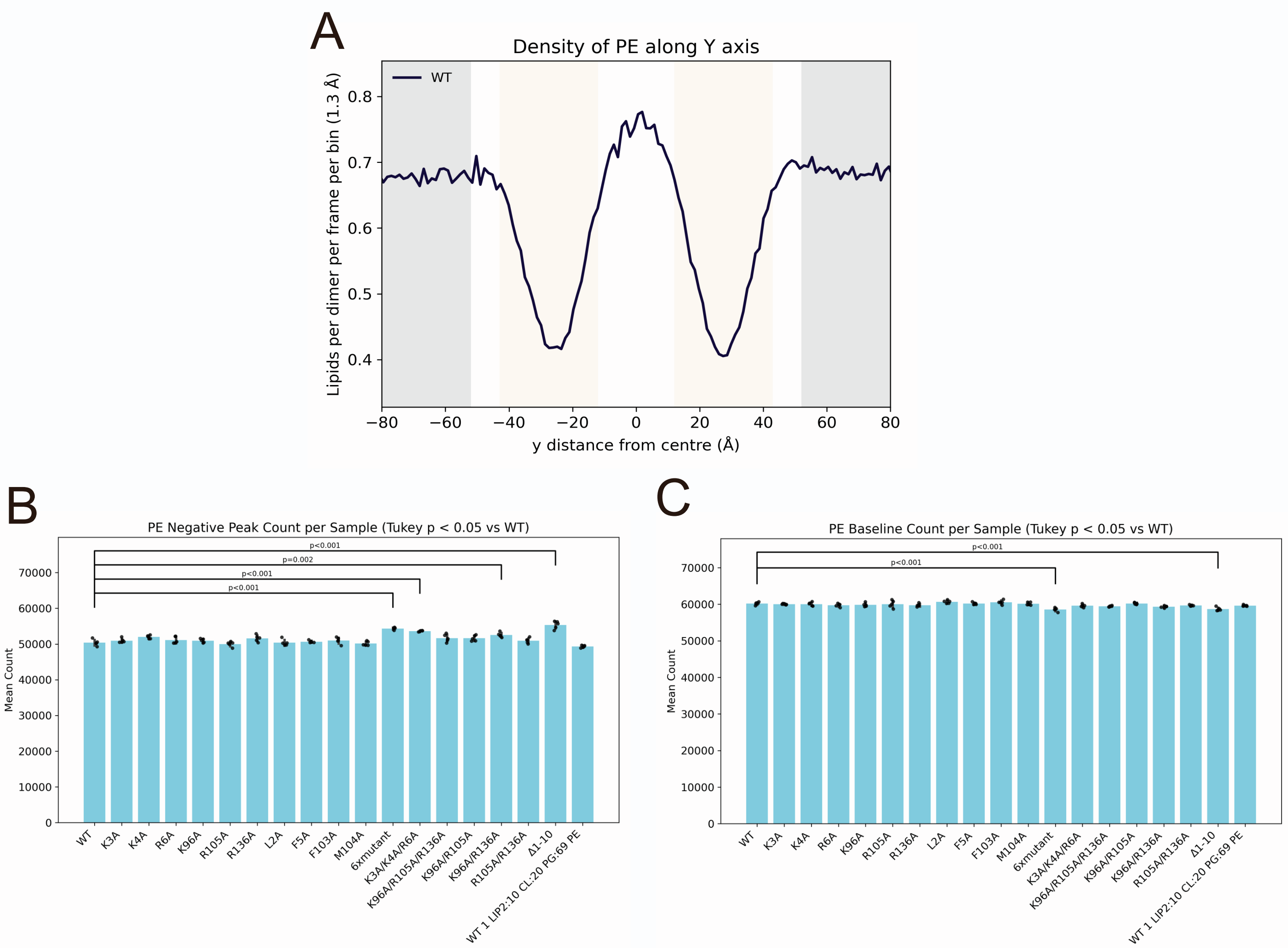

Figure S9: PE enrichment measurements in different conditions. A) Definition of regions on the y-axis that are compared in the lipid enrichment measurements: peach = "PE Negative Peak" (B), grey = "PE Baseline" (C). Data shown in (A) is from WT MreB with 1 CL: 2 PG: 7 PE membrane. B-C) Count of PE molecules in the regions defined in (A) in different mutants and simulation conditions. Significant differences between WT and other conditions are labelled with their p-value from an ANOVA and Tukey-HSD test.

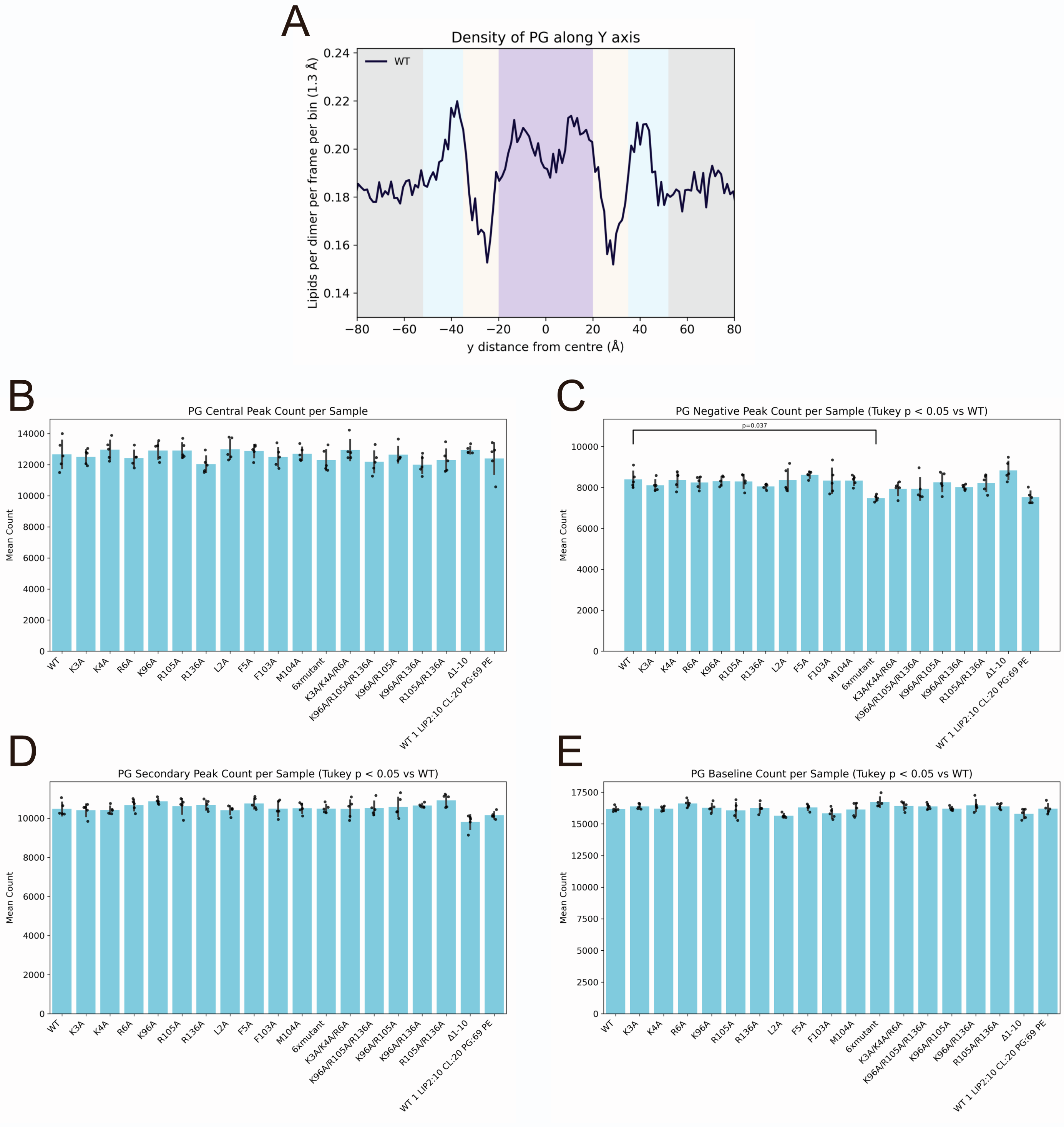

Figure S10: PG enrichment measurements in different conditions. A) Definition of regions on the y-axis that are compared in the lipid enrichment measurements: purple = "PG Central Peak" (B), peach = "PG Negative Peak" (C), pale blue = "PG Secondary Peak" (D) grey = "PG Baseline" (E). Data shown in (A) is from WT MreB with 1 CL: 2 PG: 7 PE membrane. B-D) Count of cardiolipin molecules in the regions defined in (A) in different mutants and simulation conditions, all with 10% CL in the membrane. Significant differences between WT and other conditions are labelled with their p-value from a Tukey-HSD test ($p<0.05$). PG Central Peak (B) had $p>0.05$ in ANOVA so Tukey HSD is not applicable.

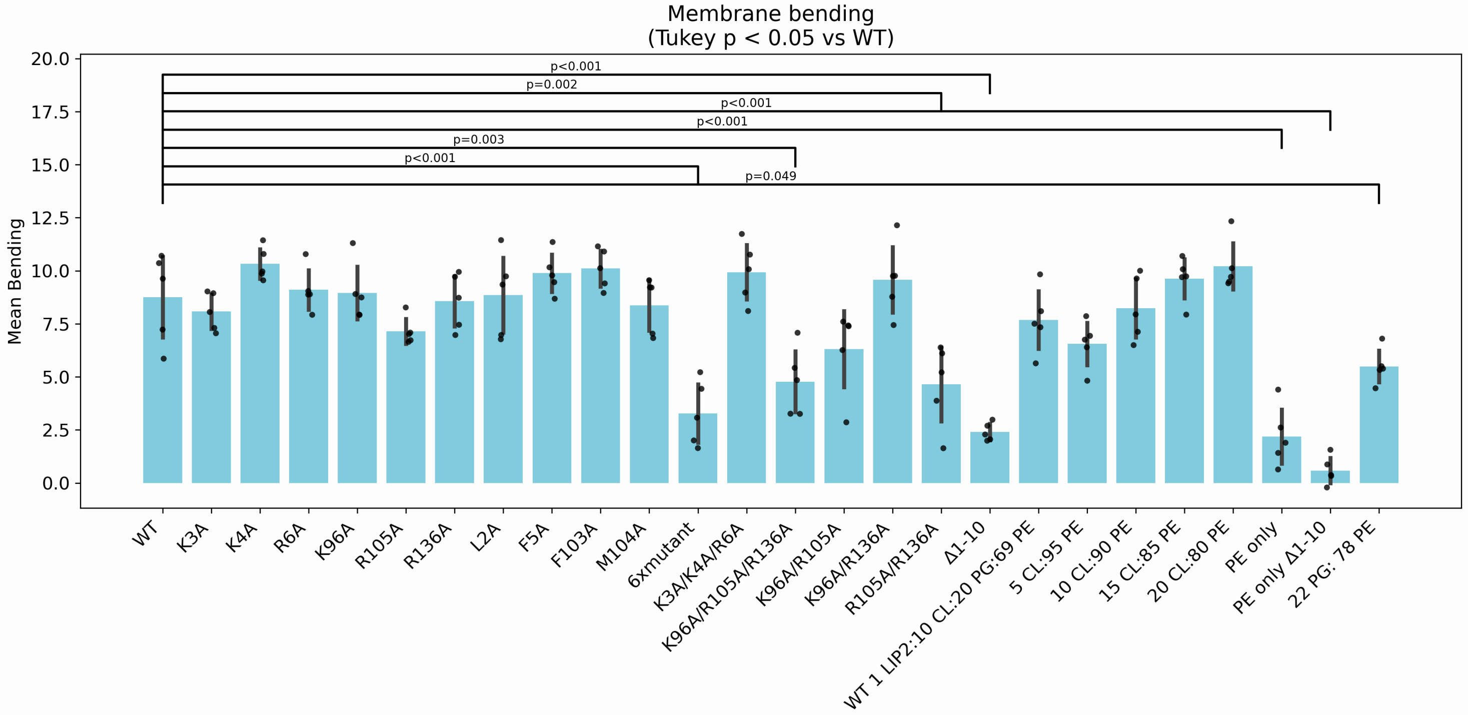

Figure S11: Membrane bending in different conditions. Significant differences between WT and other conditions are labelled with their p-value from a Tukey-HSD test ($p<0.05$).

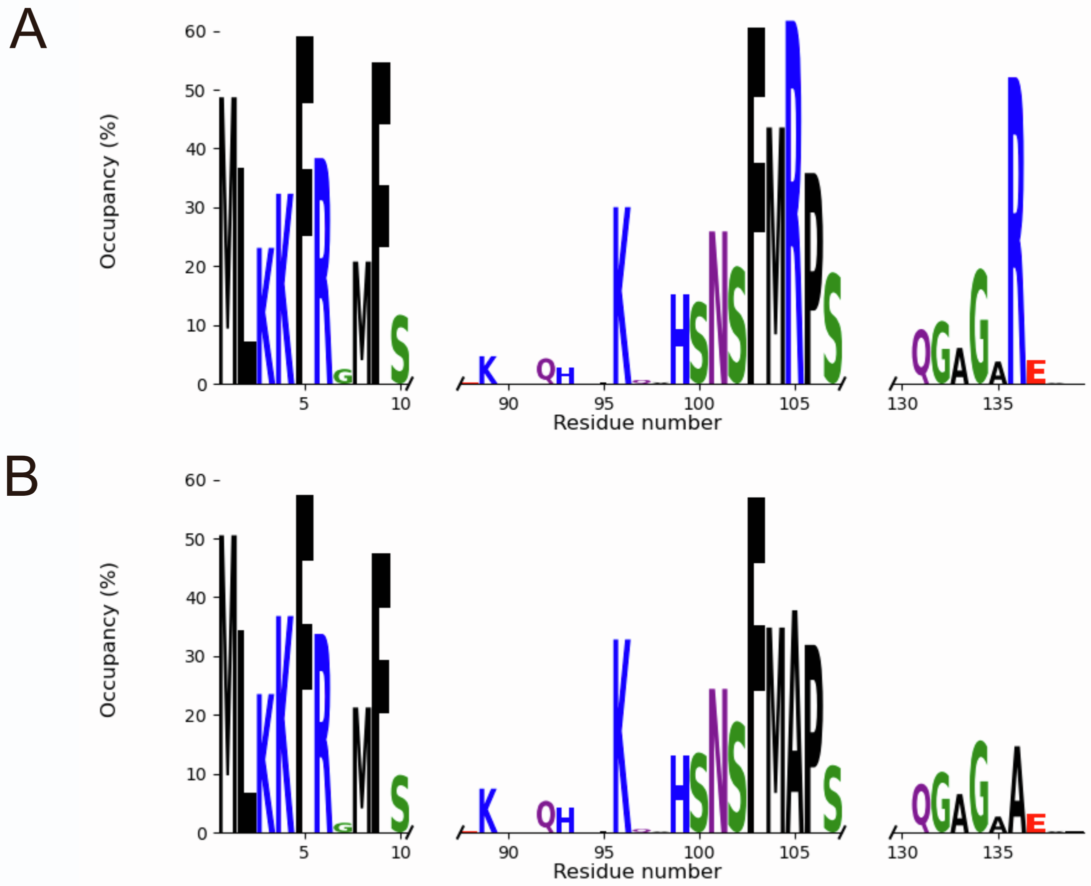

Figure S12: MreB filament interactions (occupancy) with cardiolipin molecules (percentage of the simulation during which each residue is interacting with CL). Data shown is mean per MreB monomer across the filament. A) WT MreB filament with 1 CL: 2 PG: 7 PE membrane. B) R105A/R136A mutant MreB filament with 1 CL: 2 PG: 7 PE membrane. Residues are coloured by chemical properties: hydrophobic amino acids are black, basic are blue, acidic are red, and polar are green.

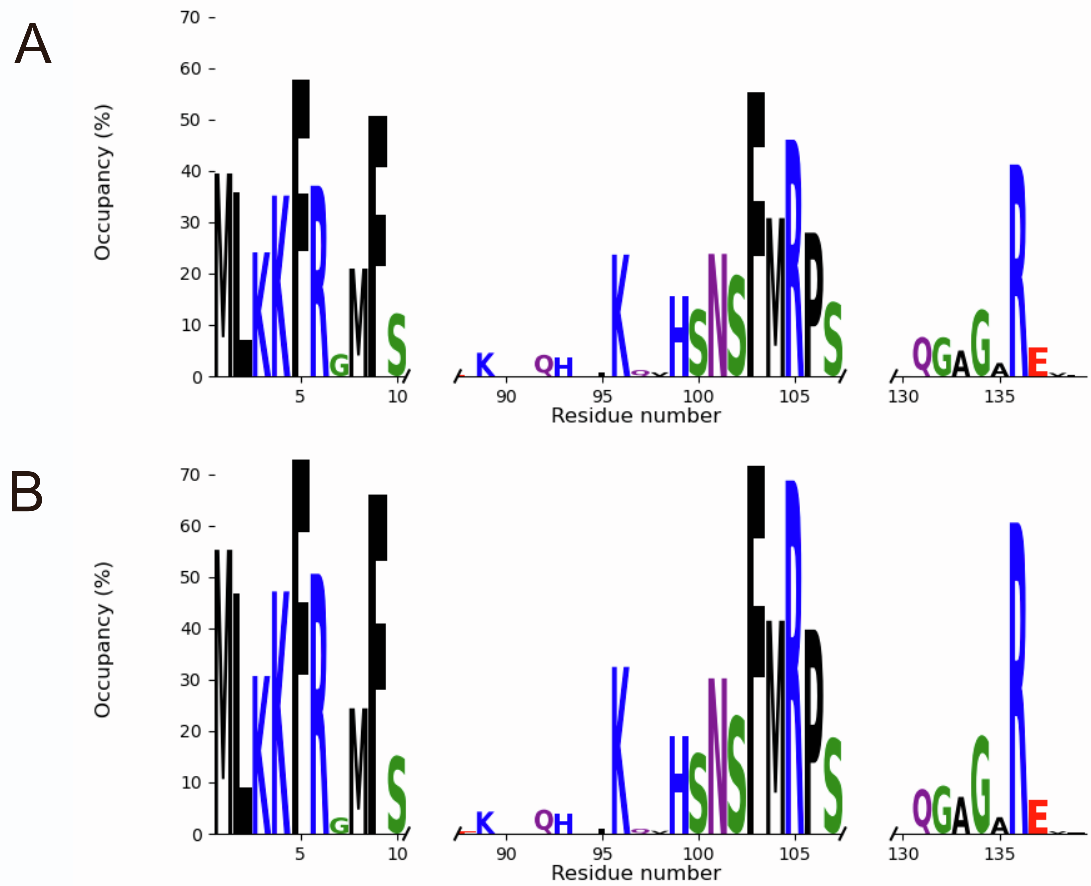

Figure S13: MreB filament interactions (occupancy) with PG molecules (percentage of the simulation during which each residue is interacting with PG. Data shown is mean per MreB monomer across the filament. A) WT MreB filament with 1 CL: 2 PG: 7 PE membrane. B) WT MreB filament with 22 PG: 78 PE membrane. Residues are coloured by chemical properties: hydrophobic amino acids are black, basic are blue, acidic are red, and polar are green.

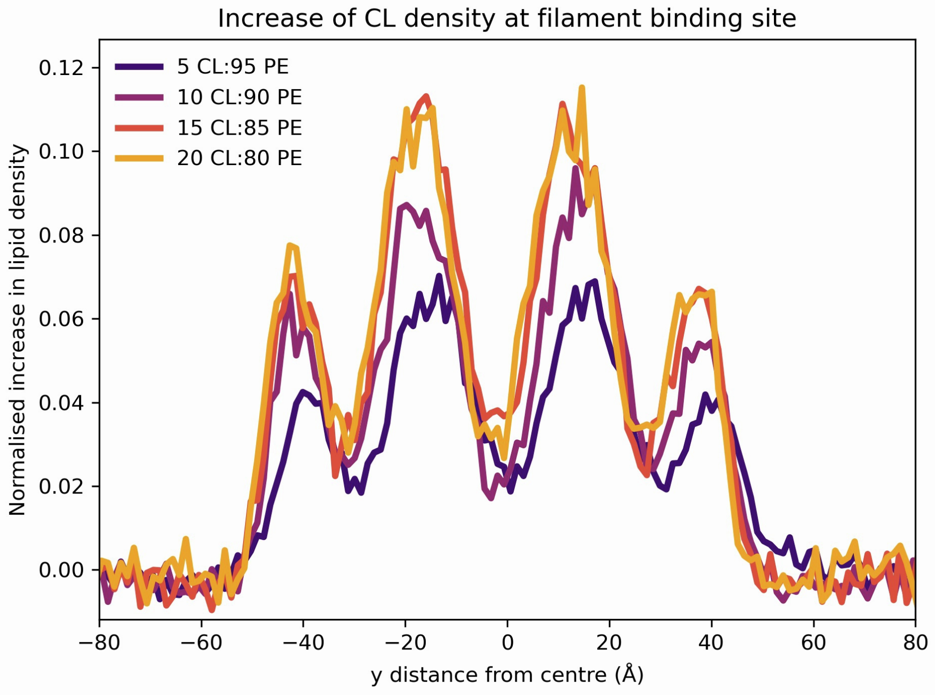

Figure S14: Increase in CL density at the MreB binding site. Normalised increase in lipid density is calculated as increase in lipids per MreB dimer per frame per 1.3 Å bin on the y axis, compared to average lipid density away from the MreB binding site.

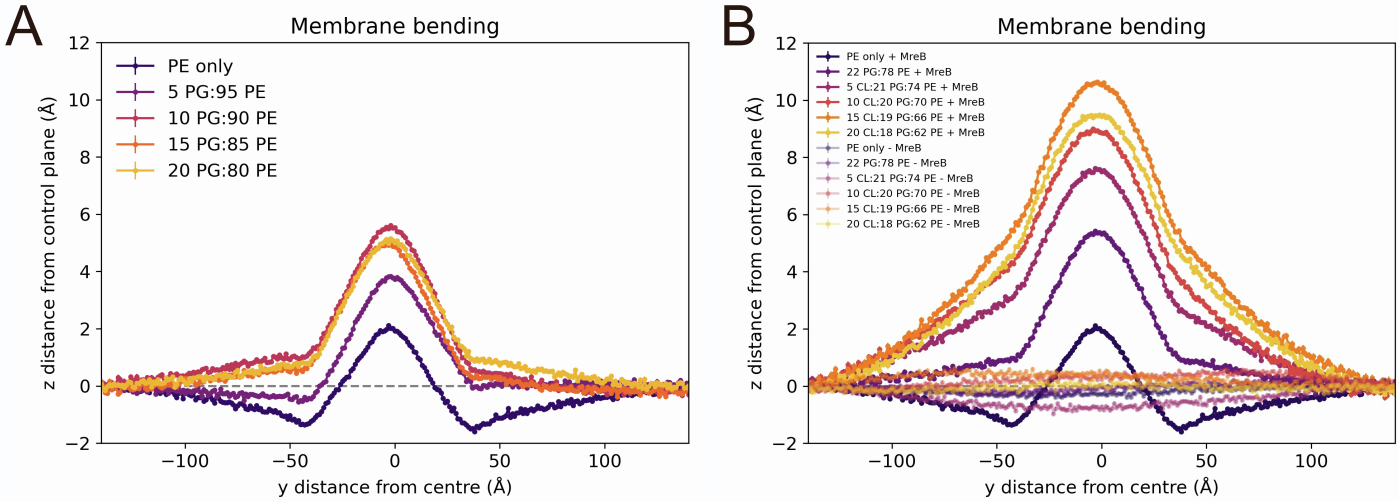

Figure S15: A) Bending of membrane by WT MreB filaments with varied PG concentrations in the membrane. The rest of the membrane is PE. B) A comparison of membrane bending at different CL concentrations in the membrane with or without MreB present.

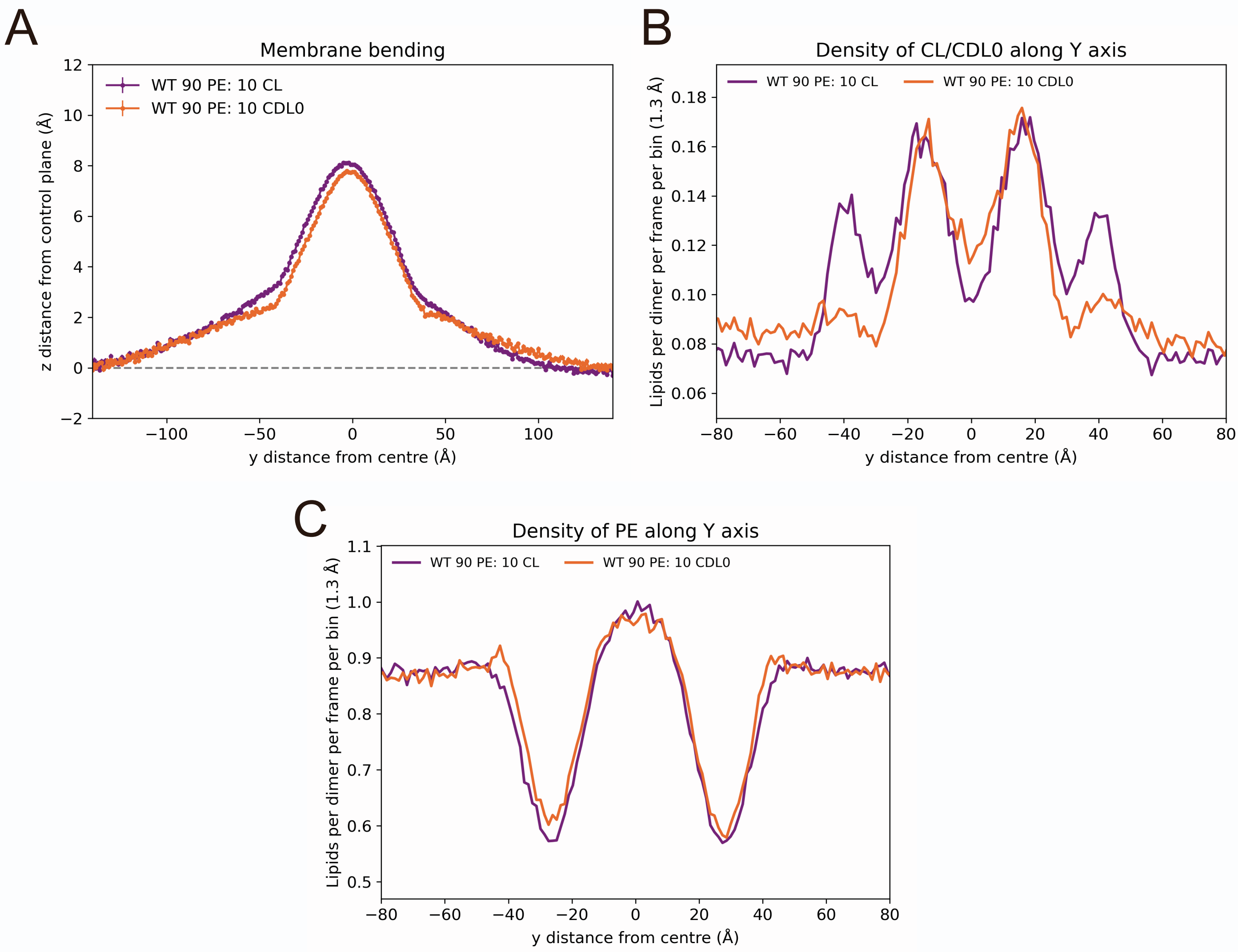

Figure S16: A) Bending of membrane by WT MreB filaments with 90% PE and either 10% CL or neutral CL (CDL0). B) CL/CDL0 and C) PE density in simulations with MreB filaments with 90% PE and either 10% CL or CDL0.

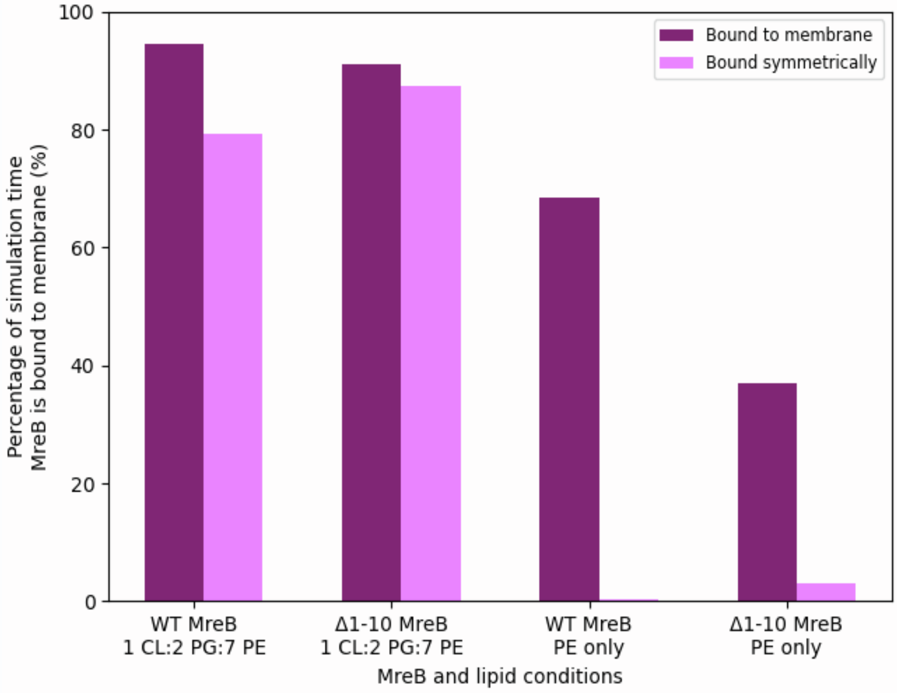

Figure S17: Association of MreB dimers to the membrane. Simulations have either 1 CL: 2 PG: 7 PE or PE-only membrane, with either WT MreB or MreB missing the N-terminal helix (Δ1-10).

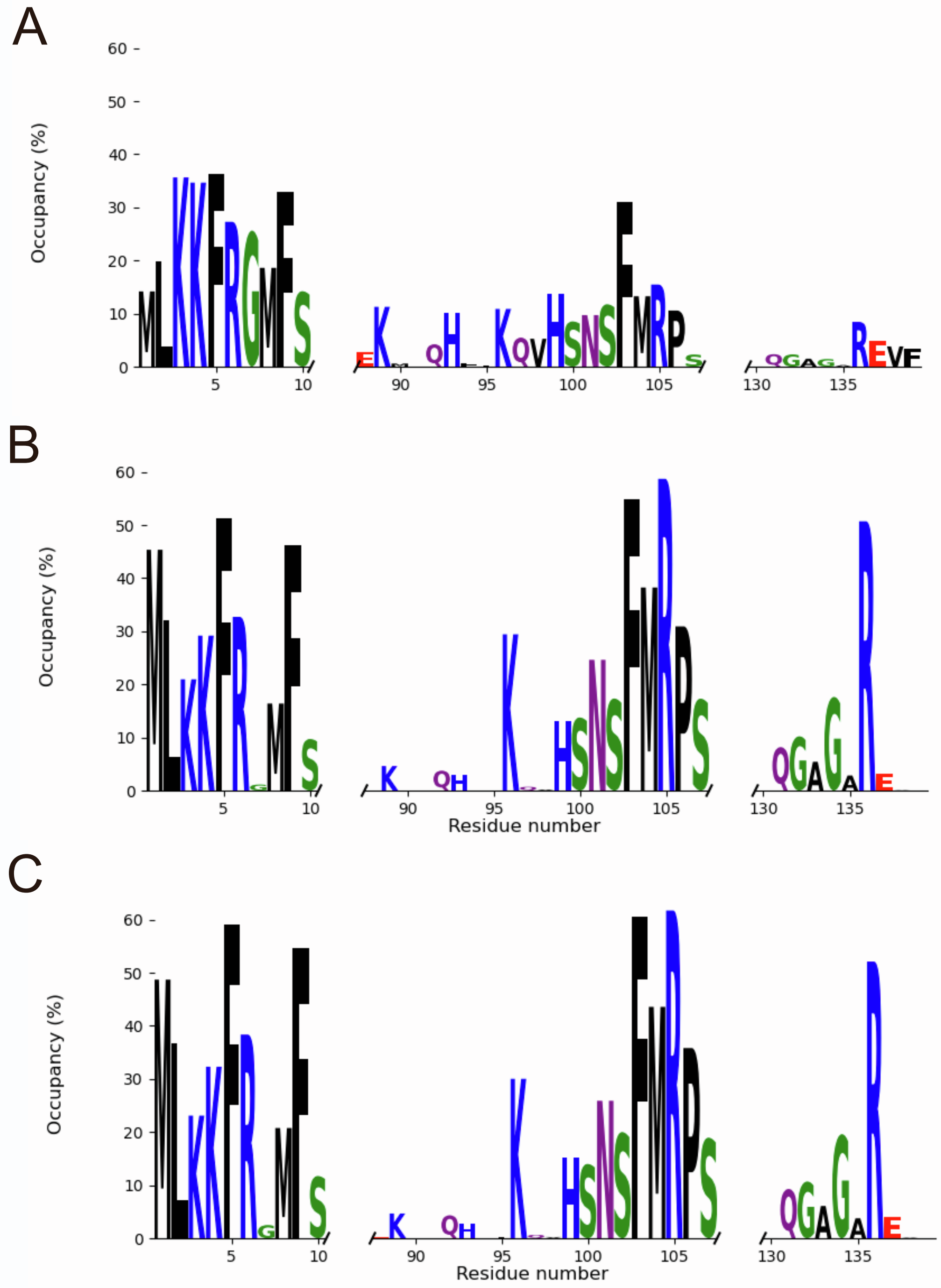

Figure S18: MreB filament interactions (occupancy) with lipid II or CL in systems with or without lipid II. Data shown is mean per MreB monomer across the filament. A) Lipid II occupancy in WT MreB filament with 1 LIP2: 10 CL: 20 PG: 69 PE membrane. B) CL occupancy in WT MreB filament with 1 CL: 2 PG: 7 PE membrane. C) CL occupancy in WT MreB filament with 1 LIP2: 10 CL: 20 PG: 69 PE membrane. Residues are coloured by chemical properties: hydrophobic amino acids are black, basic are blue, acidic are red, and polar are green.

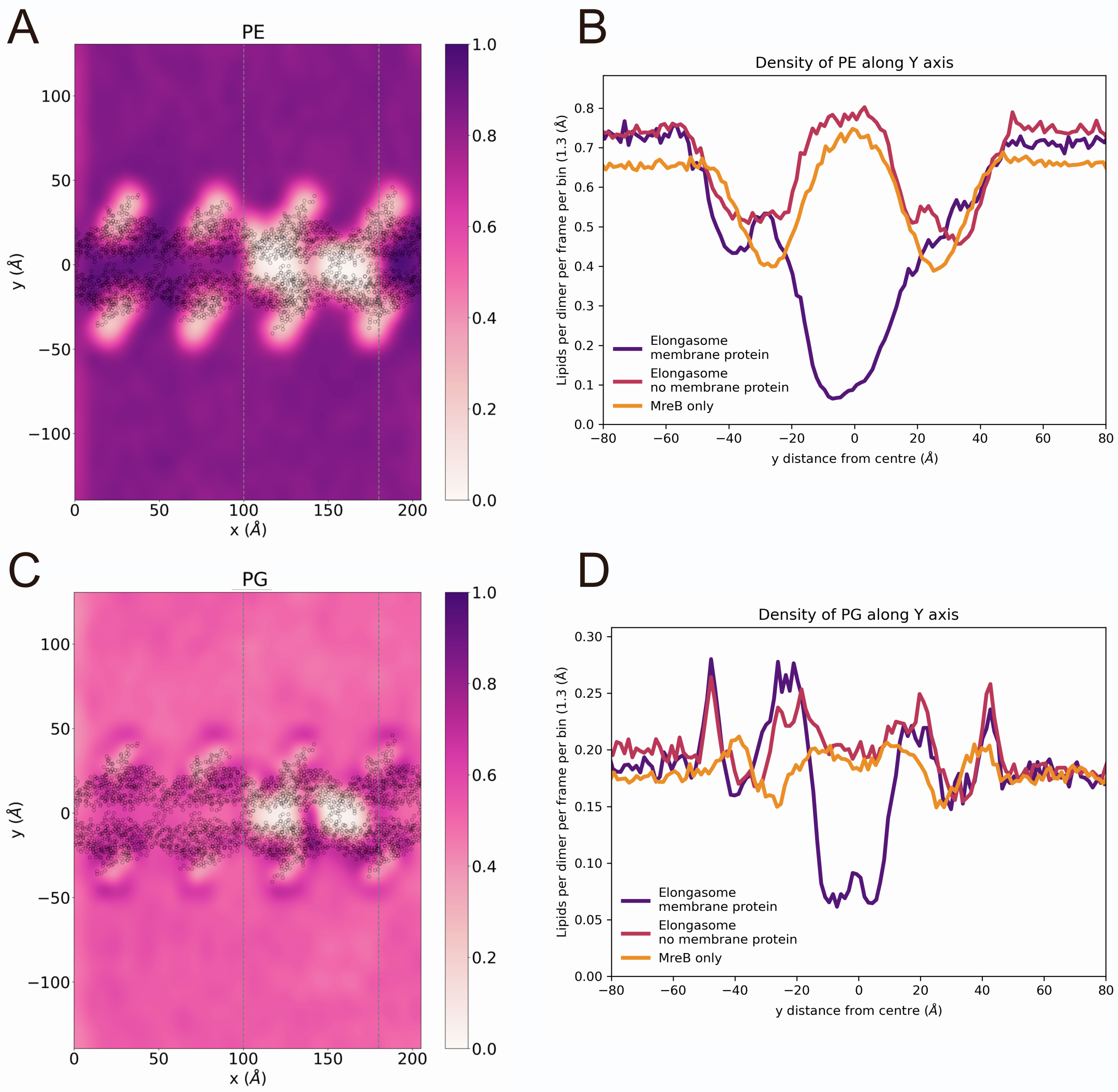

Figure S19: Density of PE (A-B) and PG (C-D) and membrane bending in elongasome simulations. A, C) 2D lipid density (colour scale) overlayed with the MreB backbone represented by grey circles. The vertical grey dashed lines represent is considered the boundaries of “Elongasome membrane protein” in plots B and D. B, D) 1D lipid density along the y axis of the simulation box. “Elongasome membrane protein” contains data from the elongasome simulation between the vertical grey dashed lines in (B), which is where the integral membrane proteins in the elongasome are situated and therefore lipid density is lower. “Elongasome no membrane protein” contains the data outside of the vertical grey dashed lines from the same simulation. ”MreB only” contains data from the control simulation of just WT MreB with a 1 CL: 2 PG: 7 PE membrane.

Supplementary references

1 Kim, H., Fábián, B. & Hummer, G. Neighbor List Artifacts in Molecular Dynamics Simulations. *Journal of Chemical Theory and Computation* **19**, 8919-8929 (2023). https://doi.org:10.1021/acs.jctc.3c00777
